# Influence of Nutrition and Maternal Bonding on Postnatal Lung Development in the Newborn Pig

**DOI:** 10.3389/fimmu.2021.734153

**Published:** 2021-08-16

**Authors:** Josephine Schlosser-Brandenburg, Friederike Ebner, Robert Klopfleisch, Anja A. Kühl, Jürgen Zentek, Robert Pieper, Susanne Hartmann

**Affiliations:** ^1^Department of Veterinary Medicine, Institute of Immunology, Centre for Infection Medicine, Freie Universität Berlin, Berlin, Germany; ^2^Department of Veterinary Medicine, Institute of Veterinary Pathology, Freie Universität Berlin, Berlin, Germany; ^3^Charité - Universitätsmedizin Berlin, corporate member of Freie Universität Berlin and Humboldt-Universität zu Berlin, iPATH.Berlin, Berlin, Germany; ^4^Department of Veterinary Medicine, Institute of Animal Nutrition, Freie Universität Berlin, Berlin, Germany; ^5^Department Safety in the Food Chain, German Federal Institute for Risk Assessment, Berlin, Germany

**Keywords:** neonate, microbiota, respiratory immunity, environment, formula, translational model, neonatal lung development

## Abstract

**Background:**

Microbial colonization and immune cell maturation coincide at mucosal sites and are decisive for postnatal lung development. How external factors influence neonatal pulmonary immune development is poorly understood.

**Objective:**

To elucidate the impact of key determinants in early life, nutrition, and maternal bonding, on postnatal lung maturation in a human-relevant animal model. To investigate the underlying immunological changes of impaired lung maturation and study the mechanisms of conversion.

**Methods:**

Newborn piglets were kept with or without isolation from their mothers and fed bovine milk-based infant formula or received milk of sow. Lung growth, histomorphology, respiratory immune responses, and lung microbiota were analyzed. Mother- and sow-milk-deprived piglets received maternal material or were reintroduced to the maternal environment at varying intervals to study options for reversal.

**Results:**

Formula feeding combined with isolation of newborn piglets resulted in disturbed postnatal lung maturation. Reduced lung growth correlated with dampened IL-33 expression, impaired lung myeloid cell activation, and decreased Th1 differentiation, along with diminished richness and diversity of the lung microbiota. Transfer of bacteria-enriched maternal material reversed the negative effects on pulmonary immune maturation. Early (within 3 days) but not late (within 7 days) reintroduction to the mother allowed restoration of normal lung development.

**Conclusion:**

Our findings reveal that lung growth, respiratory immunity, and microbial lung colonization in newborns depend on postnatal diet and maternal contact, and targeting these key regulators could promote lung development during this critical life stage.

**Summary:**

Disturbances in natural diet and reduced maternal contact during the neonatal period impair postnatal lung maturation. In pediatrics, timely breast milk feeding and intensive maternal bonding represent valuable intervention measures to promote early postnatal lung development.

## Highlights

- Isolation of newborns and simultaneous formula feeding interfere with lung maturation associated with impaired microbiota diversity during the neonatal phase.
- Reduced lung growth, cellular differentiation, and activation are reversible within a short time window after birth.
- Feeding donated breast milk or transfer of maternal material to isolated newborns compensates for deficits in postnatal lung maturation.

## Introduction

Early childhood, especially the neonatal period, is important for the establishment and development of the individual immune system and the microbiome (1). Here, the transfer of specific microbial taxa is evident, demonstrating the importance of maternal microbiota composition for promoting health and development in the offspring. However, since successful maternal microbiota transfer is only possible through physical closeness, breastfeeding and breast milk, this underlines the importance of mother–infant bonding for postnatal development. Among other disturbances in early life (*e.g.* cesarean section, medical intervention), dietary challenges such as formula nutrition greatly influence the microbial colonization of the gut (1–3), thereby affecting immune cell development and metabolism (4–6).

However, there is a knowledge gap regarding the effects of reduced maternal contact and dietary changes on postnatal lung maturation. After birth, the lung of the infant is immature and undergoes important developmental changes (7, 8) that are crucial for a long-term respiratory health (9–11). As recently shown, the human lower airway microenvironment changes rapidly in early life and is shaped by an interplay between the lung habitat, the developing immune system, and the formation of the microbiome (12). Based on the concept of the “neonatal window of opportunity”, the early postnatal period is assigned a critical role in lifelong host-microbial and immunological homeostasis (13). With respect to the lung, microbial colonization, immune cell development, and alveolarization coincide during this “neonatal window of opportunity”, making this early phase highly susceptible to interfering factors (10, 14). In humans, respiratory health and the development of asthma in later life have been linked to changes in environmental and nutritional conditions during the neonatal period (15–18). However, studies in humans investigating early changes of lung development are restricted due to ethical reasons and limited access to tissue material. For human medicine, the pig represents a promising biomedically relevant animal model with important anatomical, physiological, and immunological similarities to the human respiratory tract (19–21). Ontogenetically, lung development in pigs is very similar to that of humans (8). The respiratory system in pigs is more mature at birth than those of rodents, and postnatal alveolarization is more rapidly completed (22). Thus, the pig model is particularly suitable to study early postnatal lung development and its possible influencing external factors (*e.g.* husbandry, nutrition). So far, most of the studies investigating principles of alveolarization have been conducted in rodents. At birth, the mouse lung is comparable to the lung developmental stage of premature infants (23). In contrast, advanced lung maturity of the pig at birth makes it particularly well suited for modeling postnatal lung development in term infants.

To date, there is no effective non-invasive treatment to promote lung growth and maturation after birth that provides sustained support for subsequent lung health. Currently, treatments targeting postnatal lung development mostly rely on invasive procedures and drug applications such as corticosteroid administration, which can be associated with significant side effects (24). We hypothesized that nutrition and maternal bonding, key determinants in early life, impact neonatal lung development by modulating lung growth, immunity, and microbial colonization locally in the airways. We also put forward the hypothesis that the adverse effects of infant formula feeding in an environment without maternal contact could be mitigated by the administration of breast milk or by the transfer of maternal material and could be reversed within a certain time frame. Our data demonstrate profound negative effects of formula feeding on postnatal lung maturation in sow-deprived newborn piglets. The isolation of piglets from their mothers resulted in a reduced pulmonary Th1 differentiation, associated with a decreased bacterial diversity on the mucosal surfaces of the lower respiratory tract. Alternative feeding of sow milk and the transfer of maternal environmental material in turn mitigated the effects on delayed lung maturation. Finally, the adverse effects of formula feeding and sow deprivation on postnatal lung development were reversible within a short window after birth. Therefore, timely administration of breast milk and intensive maternal bonding could be non-invasive preventive measures to promote early postnatal lung development in pediatrics, which in turn could have long-term benefits for lung health.

## Methods

### Animals, Sampling, and Necropsy

#### Ethical Statement

The porcine study was performed in accordance with the principles outlined in the European Convention for the Protection of Vertebrate Animals used for Experimental and other Scientific Purposes and the German Animal Welfare Law. Ethical approval was obtained from the State Office of Health and Social Affairs Berlin, Germany (Landesamt für Gesundheit und Soziales Berlin, Germany) for sampling lung tissue from healthy pigs (regulation number T0002/17) and for experimental procedures (approval number G0269/16 and G0043/18).

#### Kinetics—Investigating Normal Lung Development

**Figure 1:**
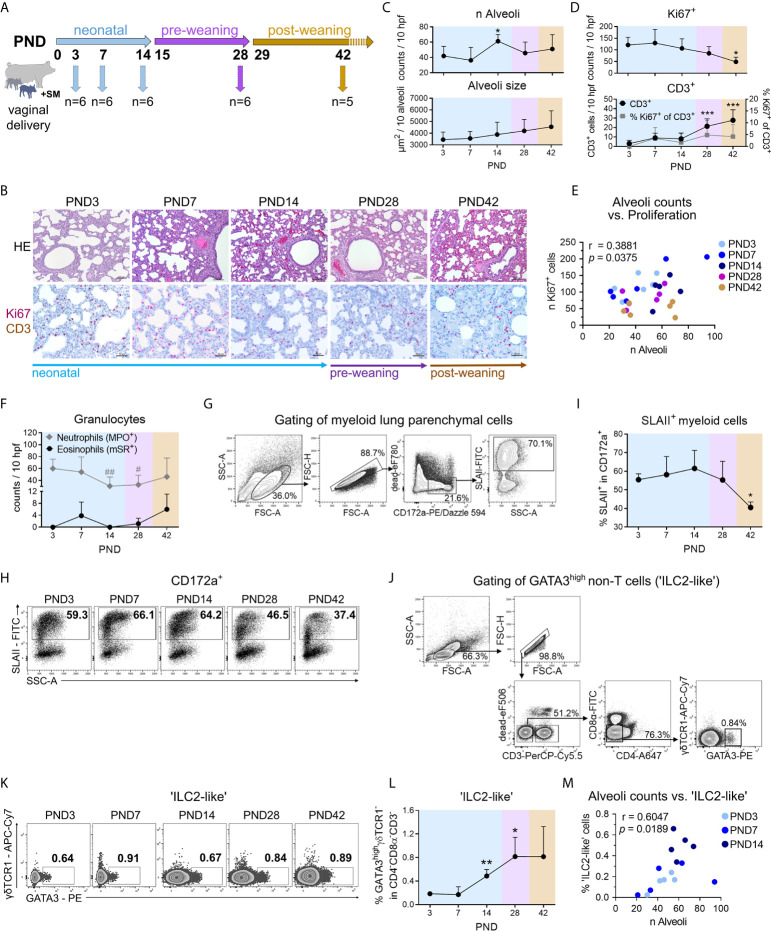
During normal postnatal lung development (PND3–PND42) in piglets, morphological changes during alveolarization are associated with increased activation of myeloid cells and recruitment of ‘ILC2-like’ cells. **(A)** Scheme of a kinetic experiment to study the postnatal lung development of conventionally reared piglets. Dissections for lung sampling and isolation of PBMC from blood were performed on PND3, 7, 14, 28, and 42 with n = 5–6 pigs per time point. **(B)** Representative hematoxylin and eosin (HE) staining [**B** (top)] and co-staining of Ki67 (purple, intranuclear) and CD3 (brown, intracytoplasmic) by immunohistochemistry [**B** (bottom)] in formalin-fixed lung tissue. **(C)** Time curve of the mean alveoli number per ten high-power fields [C (top)] and alveoli size averaged over ten alveoli [**C** (bottom)]. Data shown as mean + SD. Statistical analysis was performed by one-way ANOVA followed by Dunnett’s multiple comparisons test to a control column (=PND3). *P <.05.**(D)** Time curve of the mean number of Ki67^+^ cells [**D** (top)], CD3^+^ cells and the percentage of Ki67^+^CD3^+^ cells [**D** (bottom)] in lung tissue as determined by immunohistochemistry. Data shown as mean + SD. Statistical analysis was performed by one-way ANOVA followed by Dunnett’s multiple comparisons test to a control column (=PND3). *P <.05, ***P <.001. **(E)** Correlation of the alveoli counts and the number of Ki67^+^ lung parenchymal cells. Statistical analysis was performed by Pearson correlation (two-tailed, alpha = .05). *P <.1234. **(F)** Time curve of the mean number of granulocytes in the lung tissue, determined by immuno- and histochemical staining. The number of neutrophils is represented by MPO^+^ cells and of eosinophils by mSR^+^ cells, respectively. Data shown as mean + SD. Statistical analysis was performed by one-way ANOVA followed by Dunnett’s multiple comparisons test to a control column (=PND3). ^#^ *P* <.05, ^##^ *P* <.01. **(G)** Gating strategy to identify CD172a^+^SLAII^+^ cells (SLAII^+^ myeloid cells) in lung. **(H, I)** Representative flow cytometry plots **(H)** and time curve of mean frequencies of SLAII^+^ myeloid cells in lung **(I)**. Data shown as mean + SD. Statistical analysis was performed by one-way ANOVA followed by Dunnett’s multiple comparisons test to a control column (=PND3). *P <.05. **(J)** Gating strategy to identify lymphoid CD3^−^CD4^−^CD8*α* ^−^ *γδ*TCR1^−^GATA3^high^ (‘ILC2-like’) cells in PBMC and lung parenchymal cells. **(K, L)** Representative flow cytometry plots **(K)** and time curve of mean frequencies of ‘ILC2-like’ cells **(L)** in lung. Data shown as mean + SD. Statistical analysis was performed by Brown–Forsythe ANOVA test followed by Dunnett’s T3 multiple comparisons test to a control column (=PND3). *P <.05. **P <.01. **(M)** Correlation of the alveoli counts and the frequency of ‘ILC2-like’ cells in the lung during the neonatal phase. Statistical analysis was performed by non-parametric Spearman correlation (two-tailed, alpha = .05). **P <.0332. PND, postnatal day; hpf, high-power field; MPO, myeloperoxidase; mSR, modified Sirius Red staining.

A total of n = 29 conventionally raised crossbred landrace and large white piglets (sow-reared, suckling) were euthanized on days 3 (n = 6), 7 (n = 6), 14 (n = 6), 28 (n = 6), and 46 (n = 5) of life (**Figure 1A**). On postnatal day (PND) 30 animals were weaned and received a standard starter feed diet for piglets.

#### Experiment 1—Investigating Isolation and Formula Feeding

A total of n = 48 crossbred landrace and large white piglets with a body weight (BW) averaging 1.69 ± 0.04 kg were randomly assigned into four groups balancing for litter, body weight, and gender. Prior to separation, piglets were allowed to stay with their mothers for the first 24 h of life to take up colostrum. Treatment groups were designed as follows (see also **Figure 3A**): piglets remained with the mother receiving only maternal milk *via* suckling (SM +Sow, n = 12), piglets remained with the mother receiving maternal milk *via* suckling and in addition were fed a bovine milk based formula (SM/FO +Sow, n = 12), piglets were reared in isolators and fed with sow milk (SM −Sow, n = 12), and piglets were reared in isolators and fed with formula (FO −Sow, n = 12). Isolator reared piglets received either sow milk or the formula every 2 h between 5:00 am and 11:00 pm. Sow milk used for SM −Sow piglets was obtained by hand milking of corresponding sows during the anterior lactation and stored at −20°C until feeding. The formula was prepared to contain skimmed milk powder of cow (60%), whey powder (18%), soy oil (20%), and 2% of a mineral and vitamin premix. Body weight (BW) was determined daily, and fecal samples were collected at regular intervals. Piglets were euthanized at days 7 and 14 of life (n = 6 per group and time point).

#### Experiment 2—Investigating Backfostering

A total of n = 22 newborn crossbred landrace and large white piglets (male, female) with a BW averaging 1.51 ± 0.04 kg were randomly assigned into four different groups as follows (see also **Figure 7A**): piglets were sow-reared (S, n = 5), isolator-reared with formula for 3 days followed by back-fostering to the mother (I3, n = 6), isolator-reared with formula for 7 days followed by back-fostering to the mother (I7, n = 6), and solely isolator-reared (I, n = 5). Another additional group (n = 5) was reared isolated from the sow (I+), but additionally given a daily enrichment of their environment with a cotton cloth with which the sow and her environment were wiped off beforehand (see **Figure 6G**). Prior to separation, all piglets were allowed to stay with their mothers for the first 24 h of life to take up colostrum. The formula was ´based mainly on skimmed milk powder of cow and whey. Body weight (BW) was recorded at days 1, 3, 5, 7, 10, and 14. All animals were euthanized on day 14 of life.

#### Necropsy

Animals were sedated with 20 mg/kg BW of ketamine hydrochloride (Ursotamin^®^, Serumwerk Bernburg AG, Bernburg, Germany) and 2 mg/kg BW of azaperone (Stresnil^®^, Jansen-Cilag, Neuss, Germany). Pigs were subsequently euthanized by intracardial injection of 10 mg/kg BW of T61^®^ (Intervet, Unterschleißheim, Germany). The trachea was immediately stanched prior to tissue sample collection to avoid collapsing of the lung. For lung parenchymal cell isolation, two 2 × 2 cm sized tissue pieces of the caudal and cranial right lung lobes were placed in complete RPMI-1640 medium (PAN-Biotec GmbH) supplemented with 100 U/ml Penicillin and 100 μg/ml Streptomycin (P/S, PAN-Biotec GmbH) and stored on ice. Fecal samples and mucosal lung swabs (see **Figure 6A**) were taken and immediately snap frozen in N_2_ for DNA extraction and 16S rRNA gene amplification. Lung tissue was snap frozen in N_2_ and stored at −80°C for gene expression analysis. Additionally, lung tissue was stored in a formalin solution (Roti-Histofix 10%, Carl Roth GmbH + Co. KG) for histological examinations.

### Lung Parenchymal Cell Isolation

For isolation of lung parenchymal cells, the collected lung tissue was cut into small pieces. After washing in 20 ml of complete RPMI-1640, digestion was performed by resuspending the mashed material in RPMI-1640 containing 1% P/S, 0.125 U/ml Collagenase D (Sigma-Aldrich), 0.180 mg/ml DNaseI (Sigma-Aldrich), 0.125 mg/ml Liberase DH (Sigma-Aldrich), and 0.125 mg/ml Liberase TM (Sigma-Aldrich). The material was incubated at 37°C in a shaking water bath (250 rpm) for 2 h. After filtering (70-μm cell strainer) and washing with ice-cold HBSS (PAN-Biotech GmbH), erythrocyte lysis was performed for 5 min at room temperature. After a second filtering step (40-μm cell strainer), cells were washed with complete RPMI-1640, and the cell pellet was resuspended in complete IMDM (PAN-Biotech GmbH) supplemented with 10% FCS (PAN-Biotech GmbH), 100 U/ml Penicillin and 100 μg/ml Streptomycin (PAN-Biotech GmbH).

### Restimulation of Lung Parenchymal Cells

For detection of cytokine-producing Th cells, isolated lung parenchymal cells were rested overnight at 37°C in complete IMDM (PAN-Biotech GmbH) supplemented with 10% FCS (PAN-Biotech GmbH), 100 U/ml Penicillin, and 100 µg/ml Streptomycin (PAN-Biotech GmbH) and stimulated on the following day with PMA (20 ng/ml, Sigma-Aldrich) plus ionomycin (1 µg/ml, Sigma-Aldrich) for 4 h and in the presence of BrefeldinA (3 µg/ml, eBioscience) during the last 3.5 h of restimulation.

### Flow Cytometry and Antibodies

Cells were stained with different combinations with fluorochrome-conjugated antibodies for flow cytometry analyses (BD FACS Canto II, BD FACSAriaIII, BD FACS Diva software, FlowJo v10 software by Tree Star) according to agreed standards (Cossarizza et al., 2019, EJI). The following porcine-specific antibodies were used: anti-CD3*ϵ*-PerCPCy5.5 (clone BB23-8E6-8C8, IgG2a, BD Biosciences), anti-TCR1*δ*-unlab (clone PGBL22A, IgG1, Kingfisher Biotech), anti-CD4*α*-AlexaFluor^®^ 647 or -PE-Cy7 or -PerCP-Cy5.5 (clone 4-12-4, IgG2b, BD Biosciences), anti-CD8*α*-FITC or -AlexaFluor^®^ 647 (clone 76-2-11, IgG2a, BD Biosciences) and anti-IFN-*γ*-PE or -AlexaFluor^®^ 647 (clone P2G10, IgG1, BD Biosciences), anti-CD163-FITC (clone 2A10/11, IgG1, Bio-Rad Laboratories, Inc.), anti-SLAII-unlab or -FITC (clone 2E9/13, IgG2b, Bio-Rad Laboratories, Inc.), anti-CD172a-PE (clone 74-22-15A, IgG2b, BD Biosciences), and anti-CD172a-Biotin (clone 74-22-15, IgG1, SouthernBiotech). In addition, several cross-reactive antibodies or secondary antibodies were used: anti-human CD14-Viogreen (clone Tük4, IgG2a, Miltenyi Biotec), anti-mouse/rat Foxp3-eFluor^®^ 450 (clone FJK-16s, IgG2a, Thermo Fisher Scientific), anti-human/mouse GATA3-PE (clone TWAJ, IgG2b, Thermo Fisher Scientific), anti-human/mouse Tbet-PE-Cy7 or -PE (clone 4B10, mouse IgG1, Thermo Fisher Scientific), anti-human TNF-α-Pacific Blue (clone Mab11, IgG1, BioLegend), anti-human IL-4-PE-Cy7 (clone MP4-25D2, IgG1, BioLegend), anti-human/mouse CD11b-APC-Cy7 (clone M1/70, IgG2b, BioLegend), anti-mouse IgG-Brilliant Violet 421™ (clone Poly4053, polyclonal IgG, BioLegend), anti-mouse IgG1-APC-Cy7 (clone RMG1-1, IgG, BioLegend), anti-mouse IgG1-FITC (clone M1-14D12, IgG, Thermo Fisher Scientific), and Streptavidin-PE/Dazzle™ 594 (Biolegend). Fixable viability dyes in eFluor^®^ 506 and eFluor^®^ 780 were used to exclude dead cells (purchased from Thermo Fisher Scientific). Intracellular/intranuclear antigens were stained after fixation and permeabilization of cells (Invitrogen™ eBioscience™ Foxp3/Transcription Factor Staining Buffer Set, Thermo Fisher Scientific).

### Histological Examination

Lung tissue from the caudal left lung lobe was sampled for histology and fixed in a formalin solution (Roti-Histofix 10%, Carl Roth GmbH + Co. KG) for 6 h at RT and then stored at 4°C. Fixed lung tissue was embedded in paraffin. Paraffin sections of 1–2 µm thickness were cut, dewaxed, and stained histochemically with hematoxylin and eosin (HE) for histomorphometric analysis, and grading of interstitial broadening and infiltration was performed. For immunohistochemical evaluation, lung sections were dewaxed and subjected to a heat-induced epitope retrieval step prior to incubation with anti-Ki67 (clone MIB1, Agilent Technologies) followed by incubation with biotinylated secondary antibody (donkey anti-mouse, Dianova). Biotin was detected by streptavidin coupled with alkaline phosphatase (AP) and RED as chromogen [both Dako REAL™ Detection System, Alkaline Phosphatase/RED, Mouse (Agilent Technologies)]. After chromogen development, proteins in Ki67 stained sections were inactivated followed by incubation with a cross-reactive anti-human CD3 (GA503; polyclonal rabbit, Agilent Technologies). For detection, EnVision+ System-HRP Labelled Polymer Anti-Rabbit (Agilent Technologies) was used. HRP was visualized with diaminobenzidine (Agilent Technologies) as chromogen. Nuclei were counterstained with hematoxylin (Merck Millipore) and slides coverslipped with glycerol gelatine (Merck). Negative controls were performed by omitting the primary antibody. Myeloperoxidase-positive (MPO^+^) cells were counted in 10 high power fields in histological cross sections of the lung applying a cross-reactive polyclonal Ab against human MPO (A0398; polyclonal rabbit, Agilent Technologies) and the labeled streptavidin biotin detection method. Eosinophils were detected with modified Sirius Red staining as described before (25). All slides were coverslipped with glycerol gelatine (Merck). Cells were quantified in 10 high power fields (hpf) (0.237 mm^2^) per section. Images were acquired using the AxioImager Z1 microscope (Carl Zeiss MicroImaging, Inc.). All evaluations were performed in a blinded manner.

### Quantitative Real‐Time PCR Analysis

Total RNA was extracted from lung tissue using innuPREP RNA Mini Kit 2.0 (Analytik Jena) and reverse transcribed using High Capacity RNA-to-cDNA Kit (Applied Biosystems). LightCycler^®^ 480 SYBR Green I Master Mix (Roche) was used for amplification of mRNA transcripts of *CCL2* (26), *IL33* (27) and *RPL19* (26). Efficiencies for each primer pair were determined by generating a standard curve, and mRNA expression was normalized to the housekeeping gene ribosomal protein L19 (*RPL19*). The results for the relative gene expression were calculated using efficiency-corrected 2^-ΔΔCT^ method. All amplification reactions were performed on Light-Cycler^®^ 480 II system (Roche).

### Microbiome Analysis

Total genomic DNA from lung mucosal swabs, fecal, and environmental samples was extracted using commercial kits following the instructions of the manufacturer (for fecal samples: Qiagen Stool kit, Qiagen, Hilden, Germany; for lung mucosal and environmental swabs: Macherey Nagel NucleoSpin Tissue kit, Düren, Germany, including NucleoSpin^®^ Forensic Filters for DNA recovery from swabs). Sample preparation, including amplification of the V3 to V4 regions of the 16S rRNA gene using primer set 341F-785R, equimolar mixing, sample clean-up and sequencing by Illumina MiSeq, was performed by LGC Genomics (Berlin, Germany). Demultiplexed and primer-clipped sequence data were uploaded to the MG-RAST Server (https://www.mg-rast.org/; study ID ‘formula_lung’, ‘formula_feces’, ‘formula_penfloor’, respectively) and processed by its SEED software tool. The phylogenetic profile of each sample was computed using the Green Gene reference data bank for identification. Bacterial taxa with five or less identical sequence reads per sample were removed from further analysis. Similarly, sequence reads occurring in one sample only were ignored. The remaining sequence reads were used to calculate the relative contribution of specifically assigned sequences to total sequence reads in a sample, calculation of ecological indices (Shannon diversity index, Evenness), and further statistical analyses for identified putative taxa.

### Statistical Analysis

Graphics were created and statistics were performed with GraphPad PRISM software 8.4.2 (GraphPad Software, USA). Normality was tested with the Shapiro–Wilk test. Normally distributed data were analyzed by unpaired t-test or one-way ANOVA followed by Dunnett’s multiple comparisons test, Tukey’s multiple comparisons test or Holm–Sidak’s multiple comparisons test, and by Brown–Forsythe ANOVA test followed by Dunnett’s T3 multiple comparisons test. Nonnormally distributed data were analyzed by Mann–Whitney test or Kruskal–Wallis test followed by Dunn’s multiple comparisons test. For multiple comparisons tests values with *P* <.05 (*), *P* <.01 (**), and *P* <.001 (***) were considered to be significant. Correlation analyses were assessed by parametric Pearson correlation analysis or non-parametric Spearman correlation analysis procedure. For correlation analysis, values with *P* <.1234 (*), *P* <.0332 (**), *P* <.0021 (***) were considered to be significant.

A partial least square discriminant analysis (PLS-DA) model was used to determine the bacterial taxa that contributed to discrimination between experimental groups in Exp 1, followed by variable importance in projection (VIP) using SIMCA-P+ (Version 12.0; Umetrics, Umea, Sweden). Putative taxa with the VIP values >1 were considered most discriminatory (28). Venn diagrams illustrating unique or shared individual bacterial taxa of neonatal piglets created using Venny 2.1 (https://bioinfogp.cnb.csic.es/tools/venny/).

## Results

### Normal Postnatal Lung Development in Pigs Is Highly Dynamic and Associated With Early Changes in Morphology and Cell Composition

We first characterized postnatal lung maturation over time (neonatal phase, pre-weaning, and post-weaning) in piglets normally reared with the sow and suckled until weaning (**Figure 1A**). Histological analyses revealed distinct changes in the morphological structure of the porcine lung after birth (**Figure 1B**) with a significant increase in the number of alveoli at postnatal day (PND) 14 (**Figures 1C**, **S1A**) and a moderate but non-significant increase in alveolar size over time (**Figures 1C**, **S1A**). Immunohistochemical co-staining of Ki67 and CD3 showed significantly higher numbers of proliferating lung parenchymal cells in neonatal piglets than in weaned piglets (**Figures 1D**, **S1A**). As a characteristic of lung growth and alveolarization, the number of alveoli and the number of cell-cycle active (Ki67^+^) lung parenchymal cells correlated significantly (r = 0.3881, *P* < 0.05) during postnatal lung development (**Figure 1E**). Lung-tissue infiltrating neutrophils showed initially high numbers which dropped significantly at the end of the neonatal phase (**Figures 1F**, **S1B**). In contrast, infiltrating eosinophils demonstrated a moderate but non-significant early increase at PND7 and a late postweaning increase at PND42 (**Figures 1F**, **S1B**). Interestingly, the frequency of pulmonary SLAII expressing CD172a^+^ myeloid cells being able to present antigens to T cells (**Figure 1G**) peaked on PND14 and decreased significantly after weaning (**Figures 1H, I**). Our data thus indicated an increased activation of the mononuclear phagocyte system during the neonatal phase, which was changed into a more anti-inflammatory state as the porcine lung develops. So far, ILC2s have not been described in pigs. In mice and humans ILC2s are characterized as negative for lineage marker typically expressed on T cells, B cells, or dendritic cells and highly express the transcription factor GATA3 (29). Based on the exclusion of the porcine T cell lineage markers CD3, CD4, and CD8*α*, we analyzed live GATA3^high^ *γδ*TCR1^−^ non-T cells (**Figure 1J**), since high GATA-3 expression levels were also found in porcine extra-thymic *γδ* T cells, declining with age (30). Remarkably, pulmonary GATA3^high^ *γδ*TCR1^−^ non-T cells increased significantly until weaning (**Figures 1K, L**). This is consistent with findings in adult mice in which most ILC2s in the lung developed as tissue-resident cells in the neonatal period (31) and only very few ILCs were circulating (32). During the neonatal period, infiltration with ‘ILC2-like’ cells in the porcine lung was clearly associated with alveoli formation, characterized by a significant positive correlation of pulmonary GATA3^high^ *γδ*TCR1^−^ non-T cells and alveoli counts (r = .6047, **P <.0332) (**Figure 1M**).

Together, these data showed that postnatal lung development in newborn piglets was highly dynamic and characterized by strong cell proliferation and marked tissue remodeling during alveolarization, corresponding to the induction of innate type 2 immunity and increased activation of the pulmonary mononuclear phagocyte system.

### Th1 Cells Accumulating in the Lung Represent an Immunological Correlate of Adequate Postnatal Lung Development in Pigs

**Figure 2:**
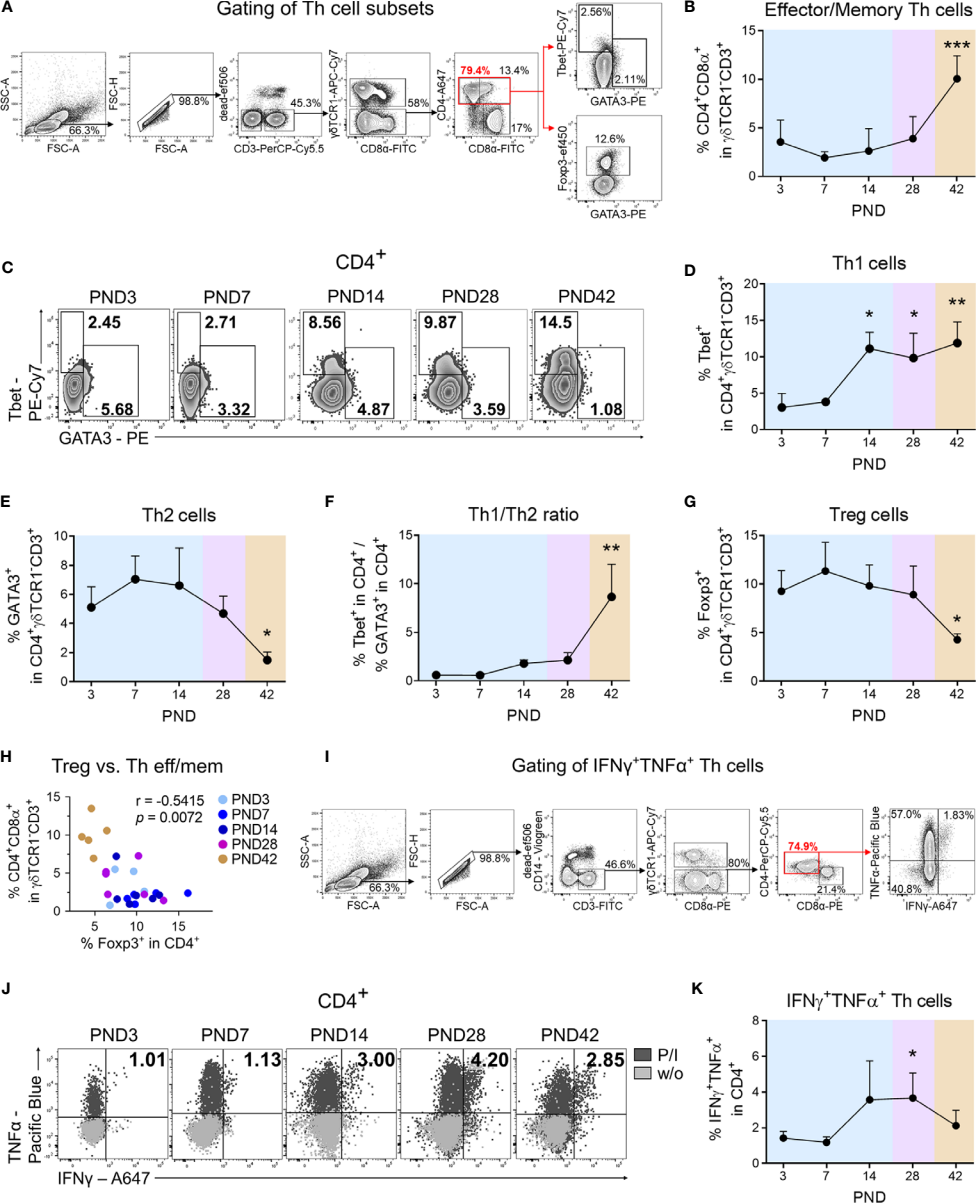
During normal postnatal lung development Th cells with effector/memory phenotype accumulate in the lung and differentiate into a type 1 phenotype. **(A)** Gating strategy to identify CD3^+^ *γδ*TCR1^−^CD4^+^CD8α^+^ cells (effector/memory Th cells), CD3^+^ *γδ*TCR1^−^CD4^+^Tbet^+^ cells (Th1 cells), CD3^+^ *γδ*TCR1^−^CD4^+^GATA3^+^ cells (Th2 cells), and CD3^+^ *γδ*TCR1^−^CD4^+^Foxp3^+^ (Treg cells) in the lung. **(B)** Time curve of mean frequencies of effector/memory Th cells in the lung. Data shown as mean + SD. To determine differences in effector/memory Th cell frequencies over time, statistical analysis was performed by ordinary one-way ANOVA followed by Dunnett’s multiple comparisons test to a control column (=PND3). ***P <.001. (**C–E**) Representative flow cytometry plots **(C)** and time curve of mean frequencies of Th1 **(D)** and Th2 cells **(E)** in the lung. Data shown as mean + SD. To determine differences in Th1 cell frequencies over time, statistical analysis was performed by Kruskal–Wallis test followed by Dunn’s multiple comparisons test to a control column (=PND3). To determine differences in Th2 cell frequencies over time, statistical analysis was performed by ordinary one-way ANOVA followed by Dunnett’s multiple comparisons test to a control column (=PND3). *P <.05, **P <.01. **(F)** Time curve of mean Th1/Th2 ratios in lung lymphocytes. Data shown as mean + SD. To determine differences in Th1/Th2 ratios over time, statistical analysis was performed by Kruskal–Wallis test followed by Dunn’s multiple comparisons test to a control column (=PND3). **P <.01. **(G)** Time curve of mean frequencies of Treg cells in lung lymphocytes. Data shown as mean + SD. To determine differences in Treg cell frequencies over time, statistical analysis was performed by ordinary one-way ANOVA followed by Dunnett’s multiple comparisons test to a control column (=PND3). **P <.01. **(H)** Correlation of the frequencies of Treg cells with effector/memory Th (Th eff/mem) cells in the lung. Statistical analysis was performed by non-parametric Spearman correlation (two-tailed, alpha = .05). ***P <.0021. **(I)** Gating strategy to identify CD3^+^ *γδ*TCR1^−^CD4^+^IFN*γ* ^+^TNFα^+^ cells (IFN*γ* ^+^TNFα^+^ secreting Th1 cells) in lung lymphocytes. **(J, K)** Representative flow cytometry plots **(J)** and time curve of mean frequencies of IFN*γ* ^+^TNFα^+^ Th cells **(K)** in lung. Data shown as mean + SD. To determine differences in IFN*γ* ^+^TNFα^+^ Th1 cell frequencies over time, statistical analysis was performed by Kruskal–Wallis test followed by Dunn’s multiple comparisons test to a control column (=PND3). *P <.05. *PND*, postnatal day; *P/I*, stimulation with phorbol-12-myristate-13-acetate (PMA) and ionomycin; *w/o*, unstimulated.

To determine the time course of T cell recruitment and T helper cell polarization during postnatal lung maturation in the pig, their frequencies and phenotypes in lung tissue lymphocytes were investigated (**Figure 2A**). CD marker expression in pigs has some peculiarities compared to humans, such as the expression of CD8*αα*-homodimers and SLAII (MHCII) molecules on activated/memory CD4^+^ Th cells (29). Within the first weeks of the life of a pig, T cells infiltrate into the lung (**Figure 1D**, see also **Figure S2A**), showing a highly activated phenotype characterized by increased frequencies and expression levels of SLAII (**Figure S2B**). The differentiation/activation state of porcine Th cells during postnatal development was examined by the analysis of CD8*α* co-expression on CD4^+^ T cells, and the frequency of pulmonary CD4^+^CD8α^+^ T cells (effector/memory Th cells) was found significantly increased after weaning (**Figure 2B**). For the discrimination of lung T cell differentiation we used transcription factor-based detection of functional subpopulations, which we previously established for the pig (33). The frequency of Tbet expressing CD4^+^ T (Th1) cells in the lung increased significantly during the first two weeks of life, indicating an early initiation of Th1 differentiation at mucosal sites (**Figures 2C, D**). Accordingly, the Tbet protein expression level per pulmonary CD4^+^ T cell (Tbet GMFI) increased significantly until the post-weaning phase (**Figure S2C**). The initial high frequencies of GATA3 expressing CD4^+^ T (Th2) cells (mean 5% of CD4^+^, PND3) in the lung dropped significantly after weaning (**Figures 2C, E**), consistent with the postulation of a strong Th2-biased immunological environment in the newborn lung (34). Accordingly, T helper cell differentiation during postnatal lung maturation was also reflected in the Th1/Th2 ratio, which increased significantly after weaning (**Figure 2F**). Regulatory T (Treg) cells are essential for the establishment of self-tolerance and immune homeostasis (35), and interestingly, they are highly abundant in neonatal murine lungs during alveolarization (9). Similarly, we found high frequencies of Foxp3 expressing CD4^+^ Treg cells during early postnatal lung maturation in the piglets, which decreased significantly after weaning (**Figure 2G**). Consequently, the ratio of effector/memory Th cells and Treg cells increased significantly after weaning at PND42 (**Figure S2D**), and an inverse correlation was observed between effector/memory Th cell and Treg cell frequencies (r = −.5415, ** *P* <.0332) (**Figure 2H**). To obtain temporal resolution of postnatal Th1 cytokine production in the pig, we examined IFN*γ* and TNFα expression in pulmonary Th cells after stimulation with PMA/ionomycin (**Figure 2I**). The frequency of IFN*γ* ^+^TNFα^+^ Th cells (**Figures 2J, K**) and TNFα expression levels (**Figure S2F**) in the lungs increased significantly during the late neonatal period at PND14 and before weaning at PND28. Accordingly, we demonstrated a significant positive correlation of the frequencies of Th1 cells with IFN*γ* ^+^TNFα^+^ Th cells (r = .6143, *** *P* <.0021) during postnatal lung development (**Figure S2E**). IL-4^+^TNFα^+^-producing Th cells were detectable only in low frequency, and these significantly decreased after weaning (**Figures S2G, H**).

In summary, our data demonstrate that Th2 and regulatory immune responses dominate in the porcine neonatal lung shortly after birth, with a shift in favor of effector/memory and Th1 immune responses after weaning (PND42).

### Formula Feeding of Newborn Piglets Separated From the Sow After Birth Results in Delayed Postnatal Lung Growth

**Figure 3:**
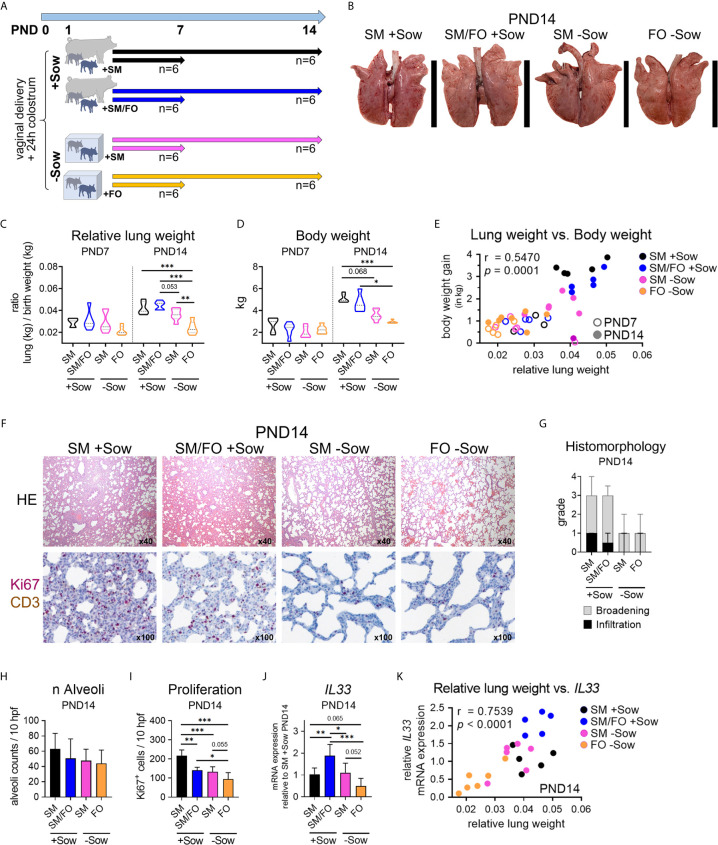
Formula feeding of newborn piglets, separated from the sow after birth, results in delayed postnatal lung growth correlated with dampened IL-33 expression. **(A)** Experimental design of a study with neonatal endpoint analysis on days 7 and 14. The piglets of each group received colostrum on the day of birth. On postnatal day 1 the animals were divided into four experimental groups as follows: Piglets were either reared with the sow and fed with sow milk (SM +Sow, n = 6 per endpoint), or were additionally given a bovine milk-based formula (SM/FO +Sow, n = 6 per endpoint), or the piglets were reared in isolation from the sow during the entire experiment and fed with sow milk (SM −Sow, n = 6 per endpoint) or with a bovine milk-based formula (FO −Sow, n = 6 per endpoint). **(B)** Representative photographs of the lung on postnatal day 14. **(C)** Relative lung weight represented as ratio of lung weight and birth weight of 7- and 14-day-old piglets. Data shown as violin plots with median and quartiles. Statistical analysis was performed by one-way ANOVA followed by Tukey’s multiple comparisons test. **P <.01, ***P <.001. **(D)** Violin plots representing the body weight of 7- and 14-day-old piglets. Data shown as violin plots with median and quartiles. Statistical analysis was performed by one-way ANOVA followed by Tukey’s multiple comparisons test. *P <.05, ***P <.001. **(E)** Correlation of the relative lung weight and the body weight gain (in kg) on postnatal days 7 and 14. Statistical analysis was performed by non-parametric Spearman correlation (two-tailed, alpha = .05). ***P <.0021. **(F)** Representative hematoxylin and eosin (HE) staining [**E** (top)] and co-staining of Ki67 (purple, intranuclear) and CD3 (brown, intracytoplasmic) by immunohistochemistry ([E (bottom)] in formalin-fixed lung tissue of 14-day-old piglets. **(G)** Bar graph demonstrating the histomorphological grading of interstitial broadening and infiltration in HE stained lung tissue of 14-day-old piglets. Bars represent the median + SD. **(H)** Bar graph representing the number of alveoli per ten high-power fields (10 hpf) in HE stained lung tissue of 14-day-old piglets. Bars represent the mean + SD. Statistical analysis was performed by one-way ANOVA followed by Holm–Sidak’s multiple comparisons test. **(I)** Bar graph representing the number of Ki67^+^ cells in lung tissue of 14-day-old piglets as determined by immunohistochemistry. Bars represent the mean + SD. Statistical analysis was performed by one-way ANOVA followed by Holm–Sidak’s multiple comparisons test. *P <.05, **P <.01, ***P <.001. **(J)** Bar graph representing the relative mRNA expression level (relative to SM +Sow PND14) of IL33 in the lung of 14-day-old piglets. Statistical analysis was performed by one-way ANOVA followed by Holm–Sidak’s multiple comparisons test. *P <.05, **P <.01, ***P <.001. **(K)** Correlation of the lung weight (in g) and the IL33 mRNA expression in lung tissue on postnatal day 14. Statistical analysis was performed by parametric Pearson’s correlation (two-tailed, alpha = .05). ****P <.0001. PND, postnatal day; SM, sow milk; FO, formula; +Sow, sow-reared; −Sow, reared without the sow.

Studies investigating early nutritional and environmental influences on postnatal lung development at the end of the neonatal period are currently lacking. Here we assessed whether isolated rearing from the sow and an altered diet impact postnatal lung maturation. One-day-old, colostrum-fed piglets were allocated into artificial rearing units and fed either sow milk or a bovine milk-based formula. In addition, conventionally sow-reared suckling piglets were fed a bovine milk-based formula. Postnatal lung development within the first 14 days of life of differently housed and fed newborn piglets were compared with normally developing sow-reared suckling piglets at PND7 and PND14 (**Figures 3A**, **S8A–G**). Isolator-rearing significantly reduced lung growth (**Figures 3B, C**, **S3A**) and body weight (**Figure 3D**) in formula-fed piglets at PND14. Accordingly, we showed a positive correlation between relative lung weight and body weight gain (r = .5470, *** *P* <.0021) during the neonatal period (**Figure 3E**). Deficits in neonatal lung growth were also reflected by histomorphological analysis (**Figure 3F**) which revealed diminished interstitial broadening and cellular infiltrates (**Figure 3G** and see also **Figure S3B**) and reduced alveoli counts (**Figure 3H**) in HE stained lung tissue of 14 days old piglets reared isolated from the sow. Immunohistochemical staining of Ki67 revealed significantly higher numbers of proliferating lung parenchymal cells in sow-reared suckling piglets compared to abnormally reared piglets at PND14 (**Figures 3F, I**). To provide further evidence of delayed alveolarization due to altered rearing conditions, we examined *IL33* gene expression in lung tissue that was decreased at PND14 in formula-fed piglets kept isolated from the sow (**Figure 3J** and see also **Figure S3C**). Interestingly, we detected a significant increase in *IL33* gene expression at PND14 in sow-reared piglets receiving an additional formula diet. Indicative of an association with the progress of postnatal lung growth, lung weight correlated significantly with *IL33* gene expression at PND14 (r = .7539, **** P <.0001) (**Figure 3K**).

Taken together, these data suggest that abnormal nutritional and rearing conditions during the neonatal period affect postnatal lung growth and that feeding breastmilk in an isolated environment mitigates the delay in postnatal lung maturation.

### Isolated Rearing in Formula-Fed Piglets Causes Deficits in the Pulmonary Mononuclear Phagocyte System

**Figure 4:**
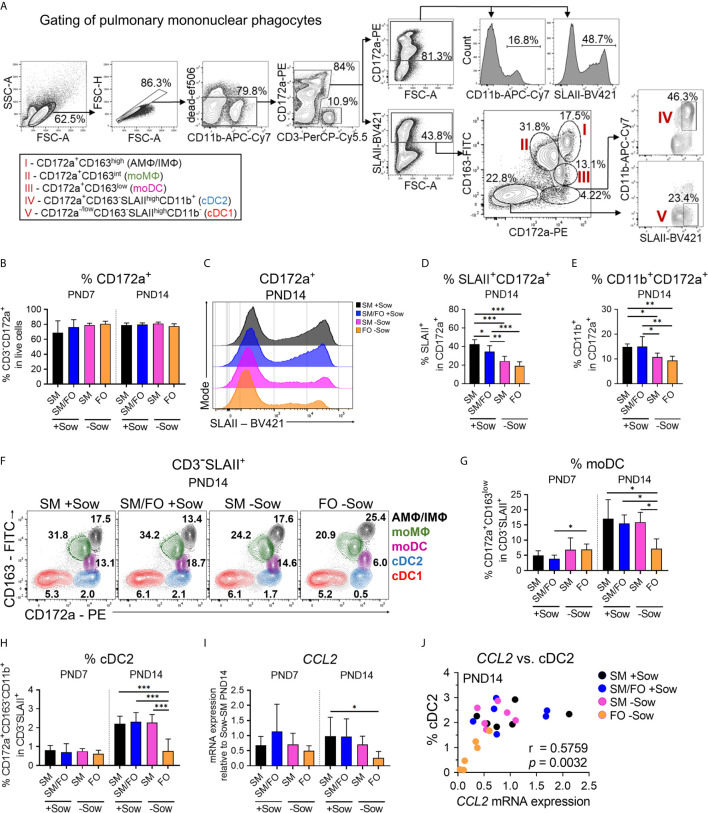
Isolated rearing in formula-fed piglets causes deficits in the pulmonary mononuclear phagocyte system that correlate with reduced CCL2 expression. **(A)** Gating strategy to identify pulmonary SLAII or CD11b expressing myeloid cells (CD3^−^CD172a^+^) and pulmonary mononuclear phagocyte subsets specified as CD3^−^SLAII^+^CD172a^+^CD163^high^ cells (AM*Φ*/IM*Φ*), CD3^−^SLAII^+^CD172a^+^CD163^int^ cells (moM*Φ*), CD3^−^SLAII^+^CD172a^+^CD163^low^ cells (moDC), CD3^−^SLAII^high^CD172a^+^CD163^−^CD11b^+^ cells (cDC2) and CD3^−^SLAII^high^CD172a^−/low^CD163^−^CD11b^−^ cells (cDC1). **(B)** Bar graph representing the frequencies of myeloid cells (CD3^−^CD172a^+^) in lung of 7-day-old and 14-day-old piglets. Bars represent the mean value + SD. Statistical analysis was performed by one-way ANOVA followed by Tukey’s multiple comparisons test. **(C, D)** Representative histograms **(C)** and bar graph **(D)** representing the frequencies of CD172a^+^SLAII^+^ cells in lung of 14-day-old piglets. Bars represent the mean value + SD. Statistical analysis was performed by one-way ANOVA followed by Tukey’s multiple comparisons test. *P <.05, **P <.01, ***P <.001. **(E)** Bar graph representing the frequencies of CD172a^+^CD11b^+^ cells in lung of 14-day-old piglets. Bars represent the mean value + SD. Statistical analysis was performed by one-way ANOVA followed by Holm–Sidak’s multiple comparisons test. *P <.05, **P <.01. **(F–H)** Representative flow cytometry plots of pulmonary mononuclear phagocyte subsets in 14-day-old piglets **(F)** and bar graphs representing the frequencies of moDC **(G)** and cDC2 **(H)** in lung of 7-day-old and 14-day-old piglets. Bars represent the mean value + SD. Statistical analyses were performed for comparison of moDC frequencies by Kruskal–Wallis test followed by Dunn’s multiple comparisons test or for comparison of cDC2 frequencies by one-way ANOVA followed by Holm–Sidak’s multiple comparisons test. *P <.05, ***P <.001. **(I)** Bar graph representing the relative mRNA expression level (relative to SM +Sow PND14) of *CCL2* in the lung of 7-day-old and 14-day-old piglets. Statistical analysis was performed by Kruskal–Wallis test followed by Dunn’s multiple comparisons test. *P <.05. **(J)** Correlation of the *CCL2* mRNA expression and the frequencies of cDC2 in the lung of 14-day-old piglets. Statistical analysis was performed by non-parametric Spearman correlation (two-tailed, alpha = .05). **P <.0332. PND, postnatal day; SM, sow milk; FO, formula; +Sow, sow-reared; −Sow, reared without the sow; AMΦ/IMΦ, alveolar macrophages/interstitial macrophages; moMΦ, monocyte-derived macrophages; moDC, monocyte-derived dendritic cells; cDC2, type 2 conventional dendritic cells; cDC1, type 1 conventional dendritic cells; GMFI, geometric mean fluorescence intensity; CCL2, CC-chemokine ligand 2.

The lung mononuclear phagocyte system is predominantly active in the neonatal period because, as part of the innate immune system, it does not require specific antigen experience (36). We therefore asked whether abnormal rearing conditions such as formula feeding and isolation from the mother affect maturation of the pulmonary mononuclear phagocyte system and whether breast milk feeding reverses adverse effects of isolation of formula-fed neonates. Identification of porcine phagocyte mononuclear subsets within lung parenchymal cells by flow cytometry analysis was adapted from Maisonnasse et al. (20) (**Figure 4A**). The frequency of total myeloid cells (CD3^−^CD172a^+^) in the lungs was not affected by different rearing and feeding conditions (**Figure 4B**). However, the expression levels of SLAII (**Figures 4C** and **S4A**) and the frequency of SLAII^+^ myeloid cells (**Figure 4D**) were significantly altered between experimental groups at PND14, with no differences at PND7 (**Figure S4B**). Similarly, the frequency of CD11b^+^ myeloid cells decreased significantly at PND14 in the lungs of piglets kept isolated from the sow (**Figure 4E** and see also **Figure S3C**). Analysis of the different lung mononuclear phagocyte subsets revealed significant changes in the frequencies of cDC2 and moDC, which were most pronounced on PND14 (**Figures 4F–H** and see also **Figures S4D–F**). Compared to sow-reared suckling piglets, formula feeding of piglets together with isolation resulted in a significant decrease in moDC (**Figure 4G**) and cDC2 frequencies (**Figure 4H**) at PND14. Interestingly, moDC and cDC2 lung frequencies were not affected in piglets receiving sow milk when kept isolated from the sow (**Figures 4G, H**).

To address what causes differential cell infiltration we investigated expression of the chemoattractant CCL2. *CCL2* expression increased over time in the lungs of sow-reared suckling piglets but was significantly reduced at PND14 in formula-fed piglets isolated from the sow (**Figure 4I**). Statistical analysis revealed a significant positive correlation between *CCL2* expression levels and cDC2 frequencies in the developing lung (r = .5759, **P <.0332) (**Figure 4J**).

Taken together, these data indicate that deficits in postnatal maturation of the pulmonary mononuclear phagocyte system of newborns can be caused by formula feeding and isolation from the mother and that these deficits can be partially compensated by feeding with breast milk.

### Reduced Pulmonary Th1 Cell Differentiation Induced by Isolation of Formula-Fed Newborn Piglets Correlates With Delayed Lung Growth and Immaturity of the Pulmonary Mononuclear Phagocyte System

**Figure 5:**
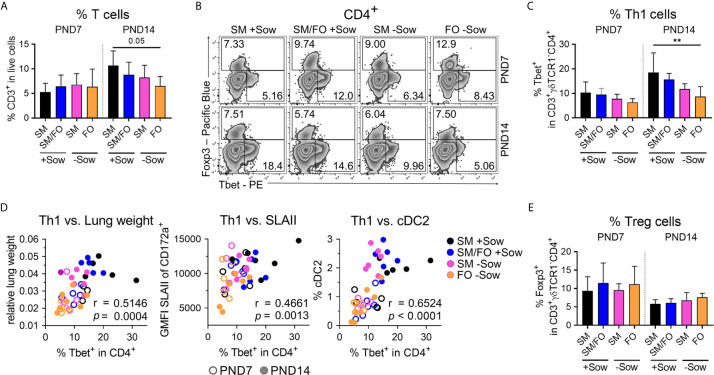
Impaired postnatal pulmonary Th1 cell differentiation program in newborn piglets isolated from the sow correlates significantly with reduced lung growth and with the immaturity of the pulmonary mononuclear phagocyte system. **(A)** Bar graph representing the frequencies of CD3^+^ T cells in the lung of 7-day-old and 14-day-old piglets. Bars represent the mean value + SD. Statistical analysis was performed by one-way ANOVA followed by Holm–Sidak’s multiple comparisons test. **(B, C)** Representative flow cytometry plots **(B)** and summarizing bar graph **(C)** representing the frequencies of Th1 cells in lung of 7-day-old and 14-day-old piglets. Bars represent the mean value + SD. Statistical analysis was performed by one-way ANOVA followed by Tukey’s multiple comparisons test. **P <.01. **(D)** Correlation of the frequencies of Th1 cells and the relative lung weight [**D** (left)], SLAII expression in CD3^−^CD172a^+^ cells [**D** (center)] and the frequencies of cDC2 [**D** (right)] in lung of 7-day-old and 14-day-old piglets. Statistical analyses were performed by parametric Pearson’s correlation (two-tailed, alpha = .05). ***P <.0021, ****P <.0001. **(E)** Bar graph representing the frequencies of Tregs in the lungs of 7-day-old and 14-day-old piglets. Bars represent the mean value + SD. Statistical analysis was performed by Kruskal–Wallis test followed by Dunn’s multiple comparisons test. *PND*, postnatal day; *SM*, sow milk; *FO*, formula; +Sow, sow-reared; −Sow, reared without the sow; *cDC2*, type 2 conventional dendritic cells.

To investigate whether altered early life conditions affect postnatal pulmonary Th1 differentiation (as shown in **Figure 2**) and the regulatory T cell response, Tbet and Foxp3 expressing Th cells were analyzed by flow cytometry in the lungs of differently kept and fed neonatal piglets. At PND14, overall T cell frequencies in the lungs were slightly reduced in formula-fed piglets isolated from sows compared to sow-reared suckling piglets (**Figure 5A**). Formula feeding in isolated piglets resulted in significantly decreased Th1 cell frequencies at PND14 (**Figures 5B, C**) and slightly decreased Tbet expression levels (**Figure S5A**), whereas the frequency of Th1 cytokine-expressing Th cells did not change significantly (**Figure S5B**). Importantly, a significant correlation was demonstrated between Th1 cell frequencies and lung weight (r = .5146, ***P <.0021), SLAII expression levels in myeloid cells (r = .4661, ***P <.0021), and cDC2 frequencies in lungs (r = .6524, ****P <.0001) at PND7 and PND14 (**Figure 5D**). Furthermore, the number of proliferating lung parenchymal cells correlated significantly with pulmonary Th1 cell frequencies (r = .7288, ***P <.0021) at PND14 (**Figure S5C**). Pulmonary Treg cell frequencies were not significantly affected by different early life conditions (**Figures 5B, E**).

These results indicate that formula feeding in an isolated environment delays postnatal pulmonary Th1 differentiation, which is also reflected in correlated impairment of lung growth and immaturity of the pulmonary mononuclear phagocyte system. Breast milk feeding attenuates these effects, which may indicate a possible mucosal cross-talk between the gut and lungs.

### Isolation of Newborn Piglets Influences the Developing Lung Microbiota, and Transfer of Bacteria-Enriched Maternal Material Supports Pulmonary Th1 Differentiation

**Figure 6:**
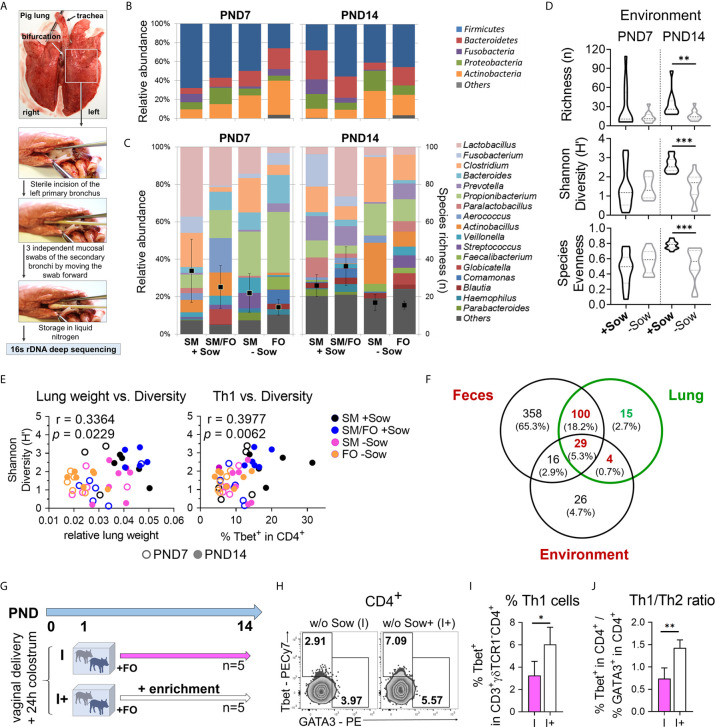
Decreased bacterial diversity correlates with a reduction in lung growth and pulmonary Th1 differentiation, which can be reversed by transfer of material from the maternal environment. **(A)** Collecting mucosal swab samples from the porcine neonatal lung to characterize the bacterial lung microbiome using 16s rDNA deep sequencing. **(B, C)** Relative abundance of major phyla **(B)**, the 17 most abundant genera and species richness (*i.e.* number of identified OTUs) **(C)** in the lungs of 7- and 14-day-old piglets. Piglets were reared with their mothers (+Sow) or in artificial rearing units without their mothers (−Sow) and received sow milk (SM) or formula (FO) as main nutrient source. **(D)** Violin plots showing richness (n), Shannon Diversity (H′) and Species Evenness of lung microbiota on days 7 and 14 of life in neonatal piglets reared in different environments (+Sow *vs.* −Sow). Statistical analysis was performed by Mann–Whitney test. **P <.01, ***P <.001. **(E)** Correlations of relative lung weight and Shannon diversity [H (left)] and Th1 cell frequencies and Shannon diversity [H (right)] in the lungs of 7- and 14-day-old piglets. Statistical analyses were performed by non-parametric Spearman correlation (two-tailed, alpha = .05). **P <.0332. **(F)** Venn diagram showing the distribution of common and unique OTUs in feces, lung, and environmental samples. A total of 503, 148, and 75 different OTUs were identified in feces, lung, and environmental samples, respectively. Shared species between different habitats and unique species in the lung are given in **Table S4**. **(G)** Experimental design of a study with endpoint analysis on day 14 after birth. The piglets of each group received colostrum on the day of birth. On postnatal day 1 the animals were divided into two experimental groups as follows: The piglets were either reared in isolation from the sow throughout the experiment and fed a formula based on bovine milk (I, n = 5 per endpoint) or they were additionally given a daily enrichment of their mother sows environment with a cotton cloth with which the sow and her environment were wiped off beforehand (I+, n = 5). **(H, I)** Representative flow cytometry plots **(H)** and bar graph **(I)** representing the frequencies of Th1 (and Th2 in **H**) cells in the lung. Bars represent the mean value + SD. Statistical analysis was performed by unpaired *t*-test. *P <.05. **(J)** Bar graph of mean Th1/Th2 ratios in the lung. Bars represent the mean value + SD. Statistical analysis was performed by unpaired *t*-test. **P <.01. PND, postnatal day; SM, sow milk; FO, formula; +Sow, sow-reared; −Sow, reared without the sow; OTUs, operational taxonomic units; I, reared without sow; I+, reared without sow with enrichment.

We used our pig model to investigate whether isolated rearing and formula feeding of newborn piglets lead to changes in the developing bacterial communities of the lungs. Mucosal swabs were taken immediately *post mortem* from the secondary bronchi to avoid contamination by species from the oral cavity and upper respiratory tract (**Figure 6A**). Rearing and feeding conditions clearly influenced the relative abundance of the major phyla (**Figure 6B**) and the 17 most abundant genera and species richness (**Figure 6C**) in the lungs of 7- and 14-day-old piglets. On PND7, *Actinobacteria* and *Bacteroidetes* predominated in piglets isolated from the sow, whereas *Fusobacteria* almost disappeared on PND14 (**Figure 6B**). At the genus level, Lactobacillus abundance was reduced in piglets reared in isolation from the sow at PND7, and Propionibacterium and Actinobacillus dominated at PND14 (**Figure 6C**). Importantly, the environment had a significant effect on species richness, Shannon diversity, and species evenness, which were reduced in sow contact-deprived piglets (**Figure 6D**). In addition, our partial least squares-discriminant analysis revealed that microbiota variations were closely clustered between normally suckled piglets and isolated but sow milk-fed piglets compared with formula-fed groups on PND14 (**Figure S6A** and **Tables S1** and **S2**). Our data indicated a significant correlation between Shannon diversity and relative lung weight (r = .3364, **P <.0332) or pulmonary Th1 cell frequency (r = .3977, **P <.0332) (**Figure 6E**). Because the inhaled or aspirated environment shapes the lung microbiota (10) and, in contrast to humans, the coprophagy of the pig contributes to microbiota transfer (37), we analyzed the shared bacterial species between different habitats (feces *vs.* environment *vs.* lung) with the majority of species present in the lungs (**Figure S6B** and **Table S3**) also detectable in the feces and environment (**Figure 6F** and **Table S4**). Accordingly, we next studied the impact of material from the maternal environment on lung immunity of sow-deprived formula-fed newborn piglets (**Figure 6G**). Th1 cell frequency (**Figures 6H, I**) and Th1/Th2 ratio (**Figures 6J**, **S6C**) were significantly increased in the group receiving material from the maternal environment, indicating improved postnatal lung development by microbial transfer.

In summary, these results suggest that isolation of neonates from the mother modifies the developing lung microbiota, with decreased richness of bacterial species correlating with reduced lung growth and Th1 differentiation. The decreased Th1 differentiation could be reversed by transferring material from the maternal environment.

### Early, but Not Late, Return to the Mother Restores Normal Lung Maturation During the Neonatal Period

**Figure 7:**
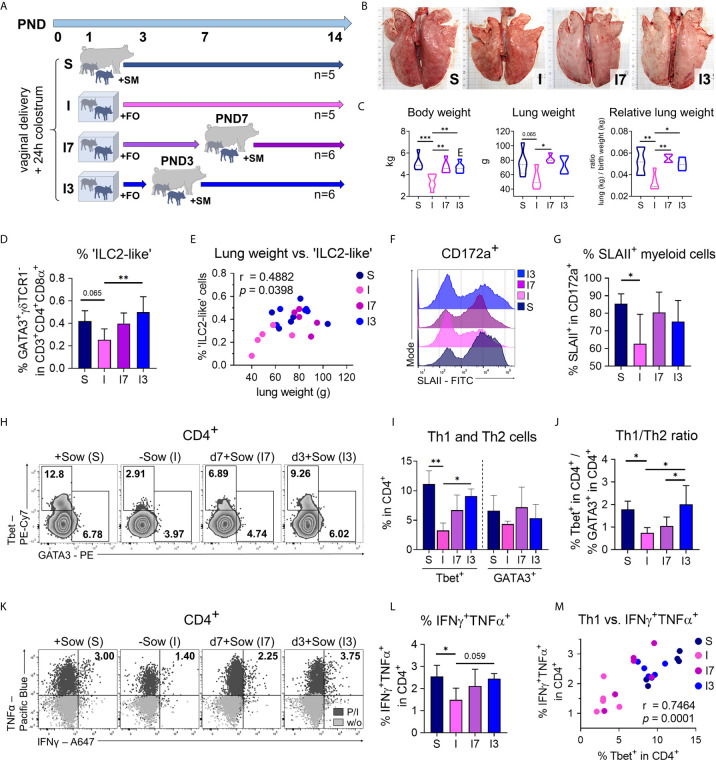
The effects of isolation and formula feeding on postnatal lung growth and immune cell development of newborn piglets are reversible within a short interval after birth. **(A)** Experimental design of the study with endpoint analysis on day 14 after birth. The piglets of each group received colostrum on the day of birth. At postnatal day 1 the animals were divided into four experimental groups as follows: Piglets were either reared with the sow and fed sow milk (S, n = 5) or piglets were reared in isolation from the sow for the entire experiment and fed a bovine milk-based formula (I, n = 5), or piglets were reared in isolation from the sow until postnatal days 7 (I7, n = 6) or day 3 (I3, n = 6) and fed a bovine milk-based formula until they were returned to the sow. **(B)** Representative photographs of the lung. The length of the black bars represents 10 cm. **(C)** Body weight [**C** (left)], lung weight [C (center)] and relative lung weight represented as ratio of lung weight and birth weight [C (right)]. Data shown as violin plots with median and quartiles. Statistical analysis was performed by one-way ANOVA followed by Tukey’s multiple comparisons test. *P <.05, **P <.01, ***P <.001. **(D)** Bar graph representing the percentage of ‘ILC2-like’ cells in the lung. Bars represent the mean value + SD. Statistical analysis was performed by one-way ANOVA followed by Holm–Sidak’s multiple comparisons test. *P <.05. **(E)** Correlation of the lung weight and the frequencies of ‘ILC2-like’ cells in lung. Statistical analysis was performed by parametric Pearson’s correlation (two-tailed, alpha = .05). *P <.1234. Twenty data points were used in total for the analysis. **(F, G)** Representative histograms **(F)** and bar graph **(G)** representing the percentage of SLAII^+^ myeloid lung parenchymal cells. Bars represent the mean value + SD. Statistical analysis was performed by one-way ANOVA followed by Holm–Sidak’s multiple comparisons test. *P < 0.05. **(H, I)** Representative flow cytometry plots **(H)** and bar graph **(I)** representing the frequencies of Th1 and Th2 cells in lung. Bars represent the mean value + SD. Statistical analyses were performed for comparison of Th1 cell frequencies by Kruskal–Wallis test followed by Dunn’s multiple comparisons test or for comparison of Th2 cell frequencies by one-way ANOVA followed by Tukey’s multiple comparisons test. *P <.05, **P <.01. **(J)** Bar graph of mean Th1/Th2 ratios in lung. Bars represent the mean value + SD. Statistical analysis was performed by one-way ANOVA followed by Tukey’s multiple comparisons test. *P <.05. **(K, L)** Representative flow cytometry plots **(K)** and bar graph **(L)** representing the frequencies of IFN*γ* ^+^TNFα^+^ Th cells (w/o correction) in lung. Bars represent the mean value + SD. Statistical analysis was performed by one-way ANOVA followed by Holm–Sidak’s multiple comparisons test. *P <.05. **(M)** Correlation of the frequencies of Th1 cells and IFN*γ* ^+^TNFα^+^ Th cells in lung. Statistical analysis was performed by parametric Pearson’s correlation (two-tailed, alpha = .05). ***P <.0021. PND, postnatal day; SM, sow milk; FO, formula; S, sow-reared; I, reared without sow; I7, reared until PND7 without sow; I3, reared until PND3 without sow; P/I, stimulated with phorbol myristate acetate and ionomycin; w/o, unstimulated.

We next asked whether the length of interfering with natural bonding and nutrition differently impacts postnatal lung maturation and the pulmonary immune system, and thus returning piglets following such an intervention to the mothers after 3 or 7 days converges the lung phenotype to normally developed piglets (**Figure 7A**). For that purpose, we investigated differences in lung growth, ILC2, and myeloid cells as well as the lung Th1 differentiation program in experimentally reared and fed groups of n = 5–6 piglets. Returning piglets to their mothers at both 3 and 7 days *postpartum* resulted in significant increases in lung growth (**Figures 7B, C**) and body weight (**Figure 7C**) compared with sow contact-deprived piglets that received formula during the entire experiment. ‘ILC2-like’ cell frequencies in the lungs were highest in piglets returned to sows 3 days after birth, with significantly elevated levels compared to sow-deprived piglets that received formula throughout the experiment (**Figure 7D**). Interestingly, lung weight and the frequency of pulmonary ‘ILC2-like’ cells were significantly correlated (r = .4882, *P <.1234) (**Figure 7E**), indicating a relationship with the progress of lung maturation. The frequency of SLAII^+^ myeloid cells was significantly reduced only in sow-deprived piglets fed formula for the entire experiment (**Figures 7F, G**). Returning piglets to their mothers on day 3 **postpartum** caused a significant increase in pulmonary Th1 cell frequencies compared to sow-deprived piglets fed formula throughout the experiment, although pulmonary Th2 cell frequencies did not change by the different conditions (**Figures 7H, I**). Compared to sow-deprived piglets fed formula throughout the experiment and piglets returned to their mothers at day 7 *postpartum*, returning piglets to their mothers at day 3 resulted in a significantly increased Th1/Th2 ratio comparable to sow-reared piglets (**Figure 7J**). Tbet expression level was significantly reduced only in sow-deprived piglets fed formula for the entire experiment, with expression levels in piglets returned to their mothers on *postpartum* days 3 and 7 approaching those of sow-reared piglets (**Figures S7A, B**). Treg cell frequencies in the lung were not affected significantly by different early life conditions (**Figure S7C**). The frequency of IFN*γ* ^+^TNFα^+^ Th cells was significantly reduced only in sow-deprived piglets fed formula for the entire experiment, and the frequency in piglets returned to their mothers on the third *postpartum* day approached that of sow-reared piglets (**Figures 7K, L**). Accordingly, we demonstrated a significant positive correlation of the frequencies of Th1 cells with IFN*γ* ^+^TNFα^+^ Th cells (r = .7464, ***P <.0021) in the lungs of differently reared newborn piglets, again demonstrating the gradual differences in Th1 differentiation (**Figure 7M**).

In conclusion, these results suggest that delayed postnatal maturation of the pulmonary immune system caused by early confounding factors, such as altered diet and separation from the mother, can be reversed within a critical window of time after birth.

## Discussion

Postnatal maturation of the respiratory immune system is highly vulnerable and requires a delicate interplay between antigenic stimulation by microbial, environmental, and nutritional factors and simultaneously requires counteracting of hyperreactivity (7, 10). However, the factors that influence early postnatal lung development and lead to the development of a particular immunophenotype and lung microbiota profile in neonates are incompletely understood. We demonstrate here for the first time that early disturbances in nutrition and maternal contact during the neonatal period significantly modulate lung maturation, respiratory immunity, and microbiome development and diversity in newborn piglets. Using the biomedically relevant large animal model, the pig, we show that decreased lung microbiota diversity correlates with reduced lung growth and Th1 differentiation in neonatal isolator-reared piglets. In addition, our data indicate that breast milk feeding and transfer of maternal material can mitigate the negative effects of formula feeding in a sow-deprived environment. Interestingly, returning previously isolated and formula-fed piglets to their mothers within a short time window after birth normalized lung growth and the pulmonary immunophenotype. Our findings highlight that nutrition and maternal bonding contribute significantly to postnatal lung development very early in the life of a newborn, and that perturbations in these factors can only be compensated for within a short time window after birth.

The immune system undergoes important compositional and functional shifts during the postnatal period and early childhood. As recently reported, shortly after birth, ILC2s, eosinophils, basophils, and mast cells spontaneously accumulate in the developing murine lung in an IL-33-dependent manner (34, 38). Mature eosinophils are transiently recruited to the neonatal murine lung during PND3–PND14, specifically corresponding to the primary septation/alveolarization phase of lung development (39). In line with this, our analysis of postnatal lung development in pigs revealed that morphologic changes during alveolarization are associated with increased activation of myeloid cells, recruitment of ‘ILC2-like’ cells, and a transient influx of eosinophils. Additionally, we demonstrated a significant correlation of *IL33* gene expression and lung weights, indicative of a relationship between alveoli formation and postnatal lung growth. Consistent with these data, previous studies in mice showed that the alveolarization phase is characterized by an early wave of IL-33 resulting in the local activation of innate type 2 immune responses (34, 38, 40). According to this, our data demonstrate that during the neonatal period in pigs, infiltration with ‘ILC2-like’ cells into the lung is clearly associated with alveoli formation. Studies in mice showed that specifically lung-resident ILC2s contribute to the phenotype and function of tissue-resident alveolar macrophages under homeostasis and promote a quiescent immune milieu (38). We revealed an inverse correlation between the frequencies of ‘ILC2-like’ cells and SLAII-expressing mononuclear myeloid cells in the developing porcine lung, indicating a comparable interdependency.

The fetal airways in humans are essentially free of lymphocytes; they are seeded after birth, with the proportion of T cells increasing in the first years of life (41). Correspondingly, our data showed that during postnatal development in pigs, T cells gradually infiltrate the lung, with Th cells with an effector/memory phenotype accumulating in the tissue and exhibiting a type 1 phenotype. Here, the Th1/Th2 ratio was initially shifted in favor of Th2 cells and increased significantly toward Th1 cells after weaning. In addition, pulmonary Tregs were found at high levels until the pre-weaning period, with a significant increase in the ratio of effector/memory T cells to Treg cells after weaning. This is in line with findings in humans and mice showing limited Th1 differentiation and enhanced Th2 and Treg differentiation during the neonatal period (42). Consistent with our data, a previous study has shown that naïve T cells predominate in human infant tissues, with effector/memory T cells restricted to mucosal sites such as the lungs (43). Supporting our data, this study showed that Treg cells constitute a high proportion of Th cells in human pediatric tissues, and the ratio of Treg cells to T effector/memory cells was lowest at mucosal sites, indicating *in situ* control of immune responses in early life. Our data highlighted that the neonatal pig is very well suited as a human-relevant model for postnatal lung maturation of term-born infants, rendering it amenable to the study of modulating *postpartum* influences.

Our experiments demonstrated a significant correlation of Th1 cell differentiation with cDC2 frequencies and microbiota richness in the lungs. In newborn mice, a particular subset of CD11c^+^CD8α^+^ IL-12-producing DCs that would allow a shift to Th1 immunity arises by day 6 after birth (44). It remains to be determined, which pulmonary DC subset in the porcine neonatal lung is likely to contribute substantially to IL-12 production and whether the reduced MHCII expression of myeloid cells we showed in sow-deprived neonatal pigs also plays a role in diminished Th1 differentiation. Recent results from the porcine model suggest that airborne bacteria make up the majority of the lung microbiome (45). Consistently, we demonstrated that the fecal and environmentally associated microbiota contribute substantially to the composition of the lung microbiome in the newborn pig, with airborne exposure likely playing a substantial role. It has recently been shown that early postnatal development of the lung microbiota is an important step in the development of tolerance to aeroallergens (9). In this context, transient induction of PD-L1 expression by CD11b^+^DCs (cDC2) and subsequent peripheral Treg cell induction played an important role in the murine model. Accordingly, we showed that formula feeding of piglets isolated from sows led to a significant decrease in pulmonary cDC2 frequency, but also resulted in a simultaneous decrease in lung microbiota diversity. However, we could not demonstrate a significant effect of postnatal environmental changes in diet and rearing on the frequencies of pulmonary Treg cells in neonatal piglets.

A number of factors contribute to adequate initial bacterial colonization in the newborn, including one important determinant, breast milk-based nutrition. Breast milk contains a variety of components that can affect intestinal mucosal immunity, as demonstrated in both humans and animal models of early life (46). The human lower airway microbiome forms within the first two postnatal months, and the mode of delivery determines the airway microbiota in preterm but not term births (12). However, data comparing the effects of alternative feeding strategies and disturbed maternal contact on postnatal lung maturation and microbial colonization in term infants are lacking. Our data showed for the first time that feeding donated breast milk to isolated newborn piglets compensates very early for deficits in postnatal lung maturation. This is in line with a very recent study showing unpasteurized breast milk to be positively associated with growth outcomes in extremely premature infants (47). Breast milk stimulates the proliferation of a balanced and diverse intestinal microbiota, which initially influences a switch from an intrauterine Th2-dominant to a Th1/Th2-balanced response and induces activation of Treg cells by breast milk-stimulated specific organisms (*Bifidobacteria*, *Lactobacillus*, and *Bacteroides*) (48). The influence of breastfeeding on lung development and function is controversial (49, 50). However, different studies showed that compared to breastfed infants, formula-fed infants had lower bacterial diversity and an altered intestinal microbiota in the first few weeks of life (51). There is emerging evidence that human milk prevents bronchopulmonary dysplasia in preterm infants, possibly by modulating the microbiome and/or inflammatory responses (52). Our analysis revealed that microbiota variations were closely clustered between normally suckled piglets and isolated but sow milk-fed piglets compared with formula-fed groups. Few studies in swine examined the microbiota composition of the lower respiratory tract, showing that reduced diversity and imbalanced microbial composition are associated with increased risk of respiratory disease (53).

Overall, our work in neonatal piglets implies that reduced maternal bonding and formula feeding can impair postnatal lung development in newborns. This is particularly important for preterm infants, with a global rate of approximately 11% (54), and other high-risk infants, with a recent study from the United States showing that 6.3% of all infants born are admitted to a neonatal intensive care unit (NICU) (55). Admission to the NICU interrupts the mother–infant bonding and the establishment of breastfeeding, resulting in lower long-term breastfeeding rates, with implications for infant health (55–57). Furthermore, our data clearly indicate that increasing the use of breast milk, whether through breastfeeding or donated breast milk, and equally important maternal contact during the early neonatal period are intervention measures to promote postnatal lung development. The non-redundant nature of postnatal immune maturation may explain the long-standing fact that early environmental exposures can have lasting consequences (58, 59). The pig, with its human-like lung physiology, served to demonstrate the importance of nutrition and bonding on lung immune maturation very early in life.

## Data Availability Statement

The microbiota dataset presented in this study can be found in an online repository. The name of the repository and accession number can be found below: https://www.ebi.ac.uk/metagenomics/, study ID PRJEB46975 (ERP131210) and study title ‘Influence of nutrition and maternal bonding on postnatal lung development’.

## Ethics Statement

The porcine study was performed in accordance to the principles outlined in the European Convention for the Protection of Vertebrate Animals used for Experimental and other Scientific Purposes and the German Animal Welfare Law. Ethical approval was obtained from the State Office of Health and Social Affairs Berlin, Germany (Landesamt für Gesundheit und Soziales Berlin, Germany) for sampling lung tissue from healthy pigs (regulation number T0002/17) and for experimental procedures (approval number G0269/16 and G0043/18). Affiliation: Landesamt für Gesundheit und Soziales (LAGeSo) Dienstgebäude Turmstr. 21 10559 Berlin.

## Author Contributions

JS-B, FE, and SH wrote the manuscript. JS-B, FE, JZ, RP, and SH designed the research approaches. JS-B, FE, and RP performed the experiments. JS-B, RP, RK, and AK analyzed data. All authors contributed to the article and approved the submitted version.

## Funding

This research was supported by the German Research Foundation (to SH: GRK 2046, HA 2542/8-1; to RP: Pi 946/2-1).

## Conflict of Interest

The authors declare that the research was conducted in the absence of any commercial or financial relationships that could be construed as a potential conflict of interest.

## Publisher’s Note

All claims expressed in this article are solely those of the authors and do not necessarily represent those of their affiliated organizations, or those of the publisher, the editors and the reviewers. Any product that may be evaluated in this article, or claim that may be made by its manufacturer, is not guaranteed or endorsed by the publisher.

## Supplementary Material

Supplementary Figure 1 Kinetic of postnatal lung development (PND3–PND42) in pigs. **(A)** Time curve of postnatal alveolarization represented by normalized data of proliferating cells (Ki67^+^), alveoli number, and alveoli size. Data shown as mean + SEM. **(B)** Representative histochemical staining for the detection of neutrophils [MPO^+^ (top)] and eosinophils [mSR^+^ (bottom)] in formalin-fixed lung tissue. PND, postnatal day; MPO, myeloperoxidase; mSR, modified Sirius Red staining.

Supplementary Figure 2 During normal postnatal lung development Th cells with effector/memory phenotype accumulate in the lung and differentiate into a type 1 phenotype. **(A)** Time curve of mean frequencies of T cells in lung. Data shown as mean + SD. To determine differences in frequencies of T cells over time, statistical analysis was performed by ordinary one-way ANOVA followed by Dunnett’s multiple comparisons test to a control column (=PND3). *P <.05, **P <.01. **(B)** Representative histograms [left] and time curve [right] of mean frequencies of SLAII^+^ T cells in lung. Data shown as mean + SD. To determine differences in frequencies of SLAII^+^ T cells over time, statistical analysis was performed by ordinary one-way ANOVA followed by Dunnett’s multiple comparisons test to a control column (=PND3). ***P <.001. **(C)** Representative histograms [left] and time curve [right] of mean Tbet expression levels in pulmonary Th cells. Data shown as mean + SD. To determine differences in Tbet expression levels over time, statistical analysis was performed by ordinary one-way ANOVA followed by Dunnett’s multiple comparisons test to a control column (=PND14). ***P <.001. **(D)** Time curve of mean Th_eff/mem_/Treg ratios in lung. Data shown as mean + SD. To determine differences in Th_eff/mem_/Treg ratios over time, statistical analysis was performed by Kruskal–Wallis test followed by Dunn’s multiple comparisons test to a control column (=PND3). ***P <.001. **(E)** Correlation of the frequencies of Th1 cells with IFN*γ* ^+^TNFα^+^ Th cells in lung. Statistical analysis was performed by non-parametric Spearman correlation (two-tailed, alpha = .05). ***P <.0021. **(F)** Representative histograms [left] and time curve [right] of mean TNFα expression levels in pulmonary Th cells. Data shown as mean + SD. To determine differences in TNFα expression levels over time, statistical analysis was performed by ordinary one-way ANOVA followed by Dunnett’s multiple comparisons test to a control column (=PND3). **P <.01. **(G, H)** Representative flow cytometry plots **(G)** and time curve of mean frequencies of IL-4^+^TNFα^+^ Th cells **(H)** in lung. Data shown as mean + SD. To determine differences in IL-4^+^TNFα^+^ Th cell frequencies over time, statistical analysis was performed by Kruskal–Wallis test followed by Dunn’s multiple comparisons test to a control column (=PND3). *P <.05. PND, postnatal day; GMFI, geometric mean fluorescence intensity; P/I, stimulation with phorbol-12-myristate-13-acetate (PMA) and ionomycin; w/o, unstimulated.

Supplementary Figure 3 Formula feeding of newborn piglets separated from the sow after birth results in delayed postnatal lung growth. **(A)** Lung weight (in g) at PND7 and PND14. Data shown as violin plots with median and quartiles. Statistical analysis was performed by one-way ANOVA followed by Tukey’s multiple comparisons test. *P <.05, **P <.01, ***P <.001. **(B)** Bar graph demonstrating the histomorphological grading of interstitial broadening [left] and infiltration [right] in HE stained lung tissue of 7- and 14-day-old piglets. Bars represent the median + SD. **(C)** Bar graph representing the relative mRNA expression level (relative to SM +Sow PND14) of IL33 in the lung of 7-day-old piglets. PND, postnatal day; SM, sow milk; FO, formula; +Sow, sow-reared; −Sow, reared without the sow.

Supplementary Figure 4 Isolated rearing in formula-fed piglets causes deficits in the pulmonary mononuclear phagocyte system. **(A)** Bar graph representing the SLAII expression levels of CD172a^+^ cells in lung of 7- and 14-day-old piglets. Bars represent the mean value + SD. Statistical analysis was performed by one-way ANOVA followed by Tukey’s multiple comparisons test. **P <.01. **(B)** Bar graph representing the frequencies of CD172a^+^SLAII^+^ cells in lung of 7-day-old piglets. Bars represent the mean value + SD. **(C)** Bar graph representing the frequencies of CD172a^+^CD11b^+^ cells in lung of 7-day-old piglets. Bars represent the mean value + SD. **(D–F)** Bar graphs representing the frequencies of AM*Φ*/IM*Φ* **(D)**, moM*Φ* **(E)**, and cDC1 **(F)** in lung of 7-day-old and 14-day-old piglets. Bars represent the mean value + SD. Statistical analyses were performed by Kruskal–Wallis test followed by Dunn’s multiple comparisons test. *P <.05. PND, postnatal day; SM, sow milk; FO, formula; +Sow, sow-reared; −Sow, reared without the sow; GMFI, geometric mean fluorescence intensity; AMΦ/IMΦ, alveolar macrophages/interstitial macrophages; moMΦ, monocyte-derived macrophages; cDC1, type 1 conventional dendritic cells.

Supplementary Figure 5 Decreased postnatal pulmonary Th1 cell differentiation in newborn piglets isolated from the sow correlates significantly with reduced lung growth and with the immaturity of the pulmonary mononuclear phagocyte system. **(A)** Bar graph representing Tbet expression levels of Th cells in the lung of 7- and 14-day-old piglets. Bars represent the mean value + SD. **(B)** Bar graph representing the frequencies of IFNγ^+^TNFα^+^ Th cells in lung of 7- and 14-day-old piglets. Bars represent the mean value + SD. **(C)** Correlation of the frequencies of Th1 cells and Ki67^+^ cells in lung of 14-day-old piglets. Statistical analysis was performed by parametric Pearson’s correlation (two-tailed, alpha = .05). ***P <.0021. PND, postnatal day; GMFI, geometric mean fluorescence intensity; SM, sow milk; FO, formula; +Sow, sow-reared; −Sow, reared without the sow; hpf, high power field.

Supplementary Figure 6 Decreased bacterial diversity correlates with a reduction in pulmonary Th1 differentiation, which can be reversed by transfer of material from the maternal environment. **(A)** Partial Least Squares Discriminant Analysis (PLS-DA) based on the evaluation of the variable importance in projection (VIP) scoring of the 20 most abundant bacterial species in the lungs of 7- and 14-day-old piglets. VIP scoring of bacterial species in the lung is given in **Table S1** and **S2**. **(B)** Venn diagram showing the distribution of common and unique OTUs in the lungs of 7- and 14-day-old piglets. A total of 132, 129, 105, and 88 different OTUs were identified in SM +Sow, SM/FO +Sow, SM −Sow and FO −Sow groups, respectively. Unique species related to environment, diet, and group are given in **Table S3**. **(C)** Bar graph representing the frequencies of Th2 cells in lung. Bars represent the mean value + SD. Statistical analysis was performed by unpaired t test. * P <.05. PND, postnatal day; SM, sow milk; FO, formula; +Sow, sow-reared; −Sow, reared without the sow; I, reared without sow; I+, reared without sow with enrichment.

Supplementary Figure 7 Effects of isolation and formula feeding on newborn piglets are reversible within a short interval after birth. **(A, B)** Representative histograms **(A)** and bar graph **(B)** representing the expression level of Tbet in pulmonary Th cells. Bars represent the mean value + SD. Statistical analysis was performed by one-way ANOVA followed by Tukey’s multiple comparisons test. **P <.01. **(C)** Bar graph representing the frequency of Treg cells in lung. Bars represent the mean value + SD. PND, postnatal day; S, sow-reared; I, reared without sow; I7, reared until PND7 without sow; I3, reared until PND3 without sow; GMFI, geometric mean fluorescence intensity.

Supplementary Figure 8 Photographs of the experimental setup. **(A)** Sow-reared piglets. **(B)** Formula-feeding of sow-reared piglets. **(C–E)** Rearing units used for sow-deprived piglets. **(F, G)** Milking of sows by hand. **(H)** Sow-deprived piglets on a cotton cloth with which the sow and her surroundings were previously wiped.

## Abbreviations

AM*Φ*, alveolar macrophage; CCL2, CC-chemokine ligand 2; cDC1, type 1 conventional dendritic cell; cDC2, type 2 conventional dendritic cell; GMFI, geometric mean fluorescence intensity; hpf, high-power field; IL-4, interleukin-4; ILC2, group 2 innate lymphoid cell; IFN*γ*, interferon gamma; IM*Φ*, interstitial macrophage; moDC, monocyte-derived dendritic cell; moM*Φ*, monocyte-derived macrophage; MPO, myeloperoxidase; MPS, mononuclear phagocyte system; mSR, modified Sirius Red stain; NICU, neonatal intensive care unit; OTUs, operational taxonomic units; PMA, phorbol-12-myristate-13-acetate; PND, postnatal day; Th cell, T helper cell; Th1 cell, Type 1 helper T cell; Th2 cell, Type 2 helper T cell; TNFα, tumor necrosis factor alpha; Treg, regulatory T cell.
