# Supplementary figures and images for: Influence of Nutrition and Maternal Bonding on Postnatal Lung Development in the Newborn Pig
Source: Front Immunol. 2021 Aug 16;12:734153. doi: 10.3389/fimmu.2021.734153 (PMC8415798; doi:10.3389/fimmu.2021.734153)

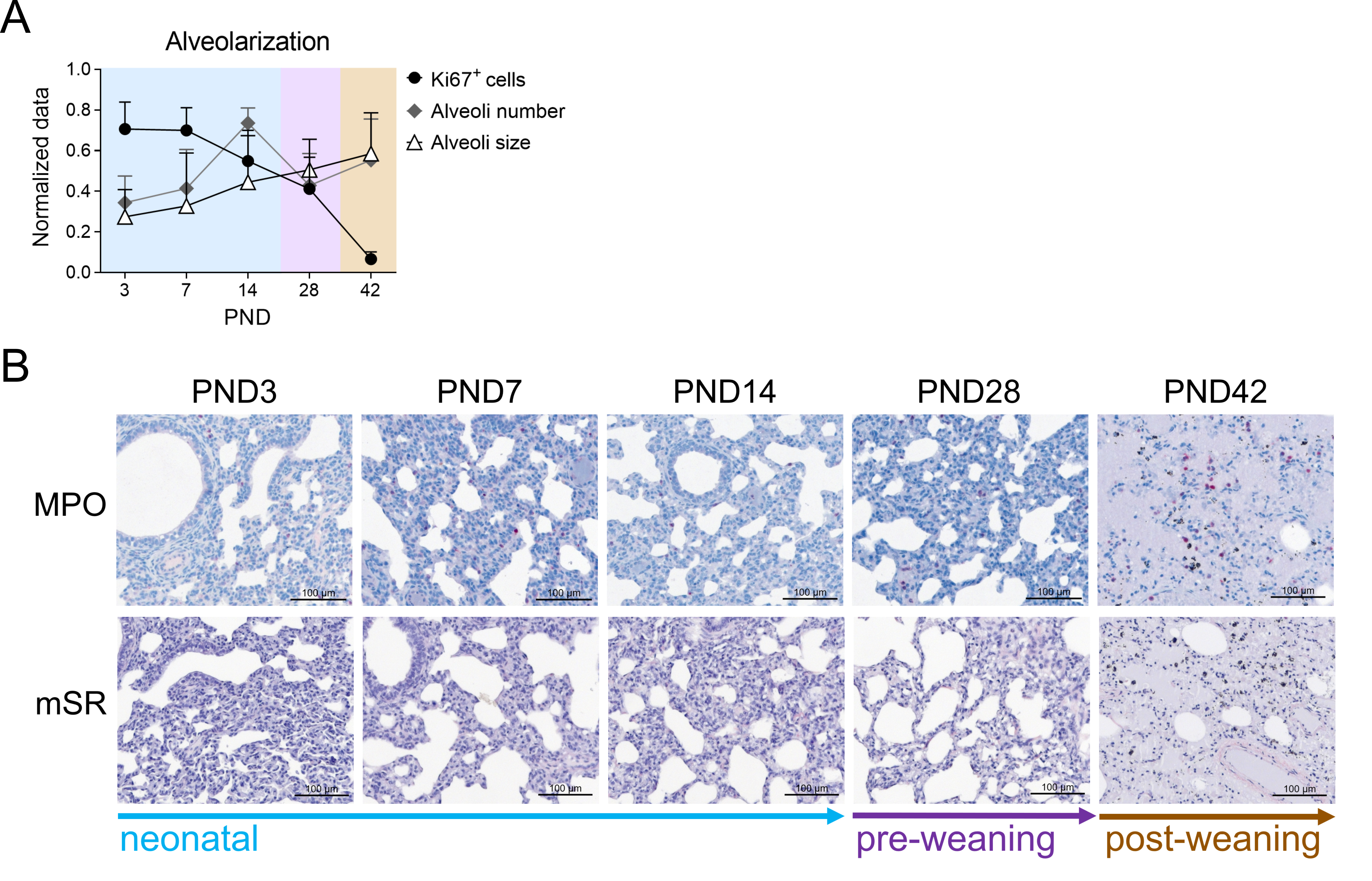

Supplement: Supplementary Figure 1 — Kinetic of postnatal lung development (PND3–PND42) in pigs. (A) Time curve of postnatal alveolarization represented by normalized data of proliferating cells (Ki67+), alveoli number, and alveoli size. Data shown as mean + SEM. (B) Representative histochemical staining for the detection of neutrophils [MPO+ (top)] and eosinophils [mSR+ (bottom)] in formalin-fixed lung tissue. PND, postnatal day; MPO, myeloperoxidase; mSR, modified Sirius Red staining. [file Image_1.tif]

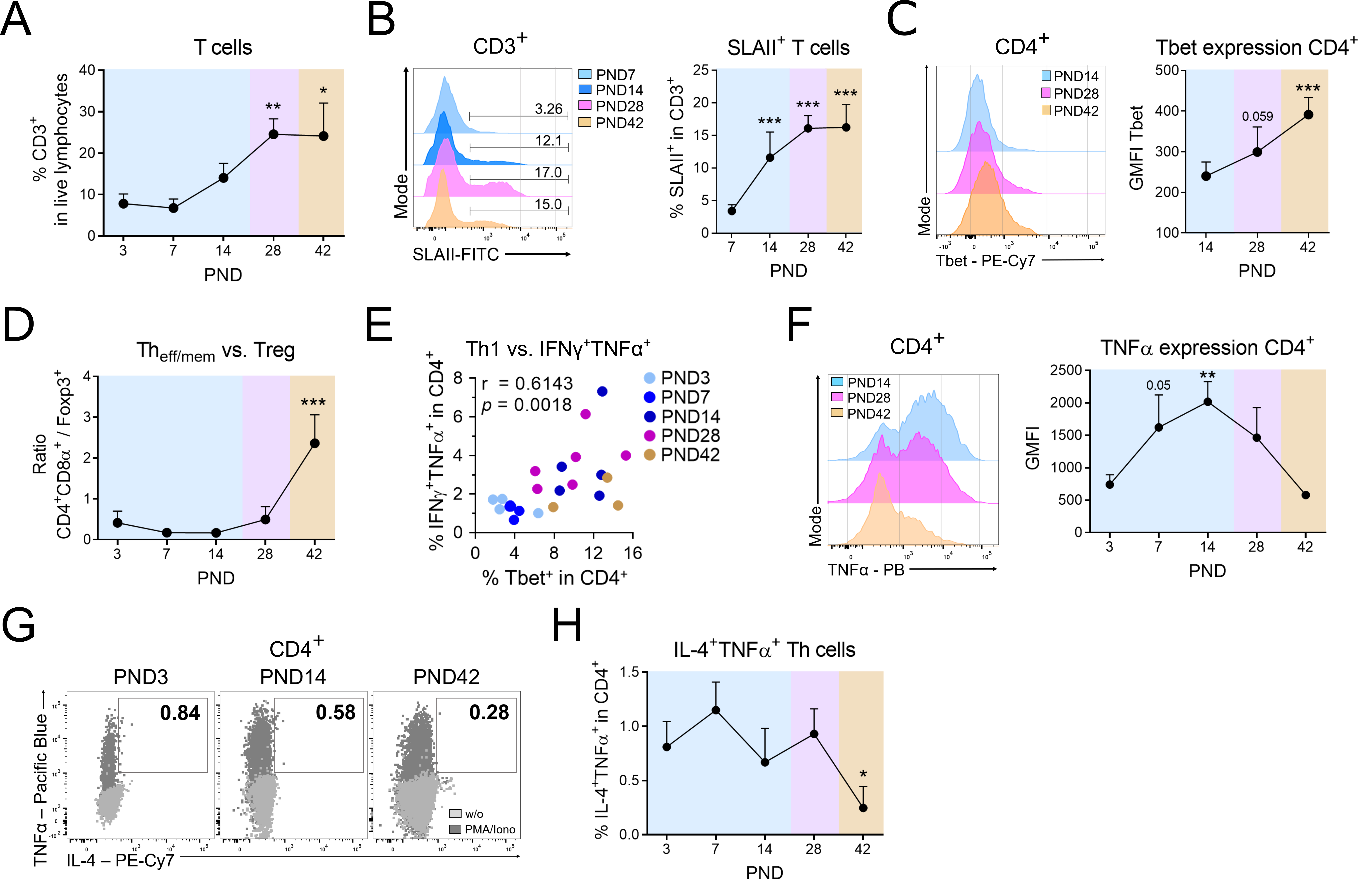

Supplement: Supplementary Figure 2 — During normal postnatal lung development Th cells with effector/memory phenotype accumulate in the lung and differentiate into a type 1 phenotype. (A) Time curve of mean frequencies of T cells in lung. Data shown as mean + SD. To determine differences in frequencies of T cells over time, statistical analysis was performed by ordinary one-way ANOVA followed by Dunnett’s multiple comparisons test to a control column (=PND3). *P <.05, **P <.01. (B) Representative histograms [left] and time curve [right] of mean frequencies of SLAII+ T cells in lung. Data shown as mean + SD. To determine differences in frequencies of SLAII+ T cells over time, statistical analysis was performed by ordinary one-way ANOVA followed by Dunnett’s multiple comparisons test to a control column (=PND3). ***P <.001. (C) Representative histograms [left] and time curve [right] of mean Tbet expression levels in pulmonary Th cells. Data shown as mean + SD. To determine differences in Tbet expression levels over time, statistical analysis was performed by ordinary one-way ANOVA followed by Dunnett’s multiple comparisons test to a control column (=PND14). ***P <.001. (D) Time curve of mean Theff/mem/Treg ratios in lung. Data shown as mean + SD. To determine differences in Theff/mem/Treg ratios over time, statistical analysis was performed by Kruskal–Wallis test followed by Dunn’s multiple comparisons test to a control column (=PND3). ***P <.001. (E) Correlation of the frequencies of Th1 cells with IFNγ +TNFα+ Th cells in lung. Statistical analysis was performed by non-parametric Spearman correlation (two-tailed, alpha = .05). ***P <.0021. (F) Representative histograms [left] and time curve [right] of mean TNFα expression levels in pulmonary Th cells. Data shown as mean + SD. To determine differences in TNFα expression levels over time, statistical analysis was performed by ordinary one-way ANOVA followed by Dunnett’s multiple comparisons test to a control column (=PND3). **P <.01. (G, H) Representative [file Image_2.tif]

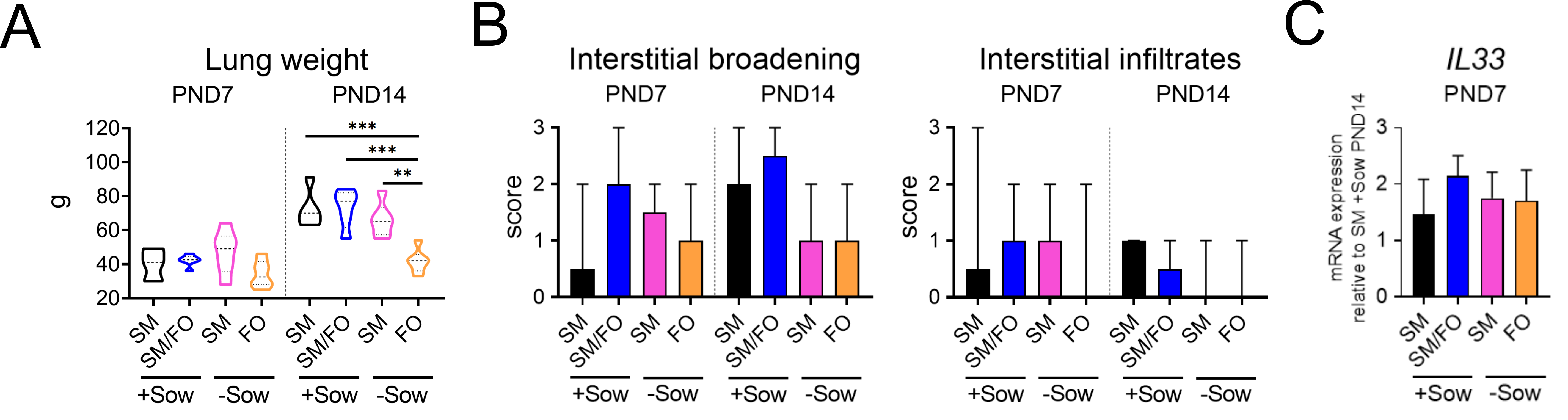

Supplement: Supplementary Figure 3 — Formula feeding of newborn piglets separated from the sow after birth results in delayed postnatal lung growth. (A) Lung weight (in g) at PND7 and PND14. Data shown as violin plots with median and quartiles. Statistical analysis was performed by one-way ANOVA followed by Tukey’s multiple comparisons test. *P <.05, **P <.01, ***P <.001. (B) Bar graph demonstrating the histomorphological grading of interstitial broadening [left] and infiltration [right] in HE stained lung tissue of 7- and 14-day-old piglets. Bars represent the median + SD. (C) Bar graph representing the relative mRNA expression level (relative to SM +Sow PND14) of IL33 in the lung of 7-day-old piglets. PND, postnatal day; SM, sow milk; FO, formula; +Sow, sow-reared; −Sow, reared without the sow. [file Image_3.tif]

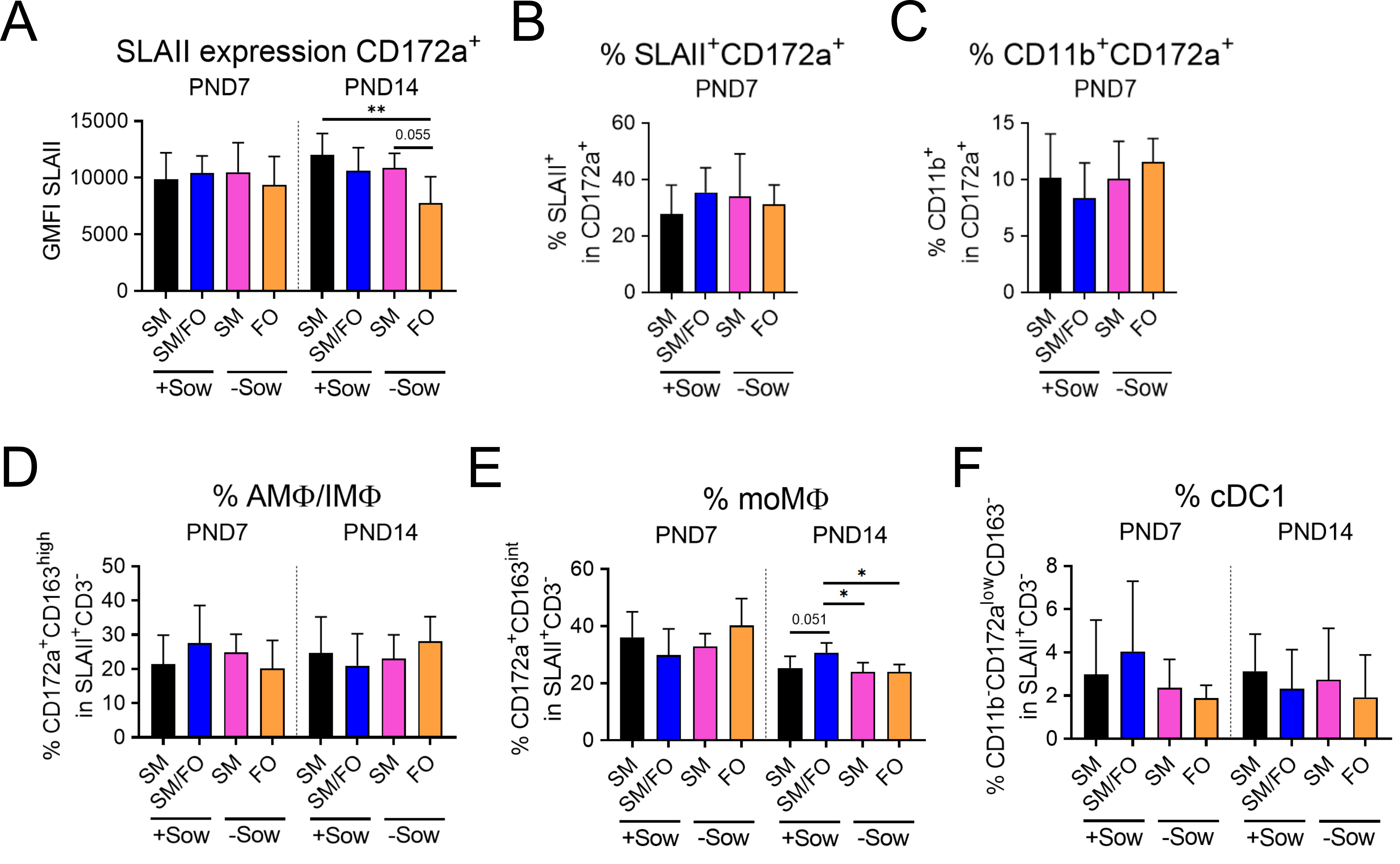

Supplement: Supplementary Figure 4 — Isolated rearing in formula-fed piglets causes deficits in the pulmonary mononuclear phagocyte system. (A) Bar graph representing the SLAII expression levels of CD172a+ cells in lung of 7- and 14-day-old piglets. Bars represent the mean value + SD. Statistical analysis was performed by one-way ANOVA followed by Tukey’s multiple comparisons test. **P <.01. (B) Bar graph representing the frequencies of CD172a+SLAII+ cells in lung of 7-day-old piglets. Bars represent the mean value + SD. (C) Bar graph representing the frequencies of CD172a+CD11b+ cells in lung of 7-day-old piglets. Bars represent the mean value + SD. (D–F) Bar graphs representing the frequencies of AMΦ/IMΦ (D), moMΦ (E), and cDC1 (F) in lung of 7-day-old and 14-day-old piglets. Bars represent the mean value + SD. Statistical analyses were performed by Kruskal–Wallis test followed by Dunn’s multiple comparisons test. *P <.05. PND, postnatal day; SM, sow milk; FO, formula; +Sow, sow-reared; −Sow, reared without the sow; GMFI, geometric mean fluorescence intensity; AMΦ/IMΦ, alveolar macrophages/interstitial macrophages; moMΦ, monocyte-derived macrophages; cDC1, type 1 conventional dendritic cells. [file Image_4.tif]

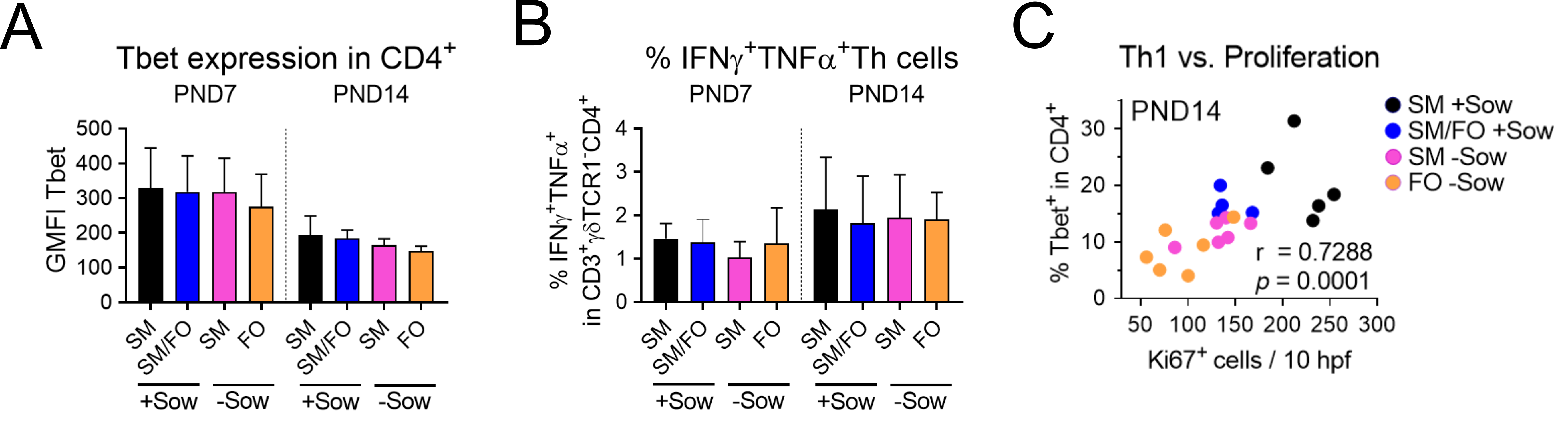

Supplement: Supplementary Figure 5 — Decreased postnatal pulmonary Th1 cell differentiation in newborn piglets isolated from the sow correlates significantly with reduced lung growth and with the immaturity of the pulmonary mononuclear phagocyte system. (A) Bar graph representing Tbet expression levels of Th cells in the lung of 7- and 14-day-old piglets. Bars represent the mean value + SD. (B) Bar graph representing the frequencies of IFNγ+TNFα+ Th cells in lung of 7- and 14-day-old piglets. Bars represent the mean value + SD. (C) Correlation of the frequencies of Th1 cells and Ki67+ cells in lung of 14-day-old piglets. Statistical analysis was performed by parametric Pearson’s correlation (two-tailed, alpha = .05). ***P <.0021. PND, postnatal day; GMFI, geometric mean fluorescence intensity; SM, sow milk; FO, formula; +Sow, sow-reared; −Sow, reared without the sow; hpf, high power field. [file Image_5.tif]

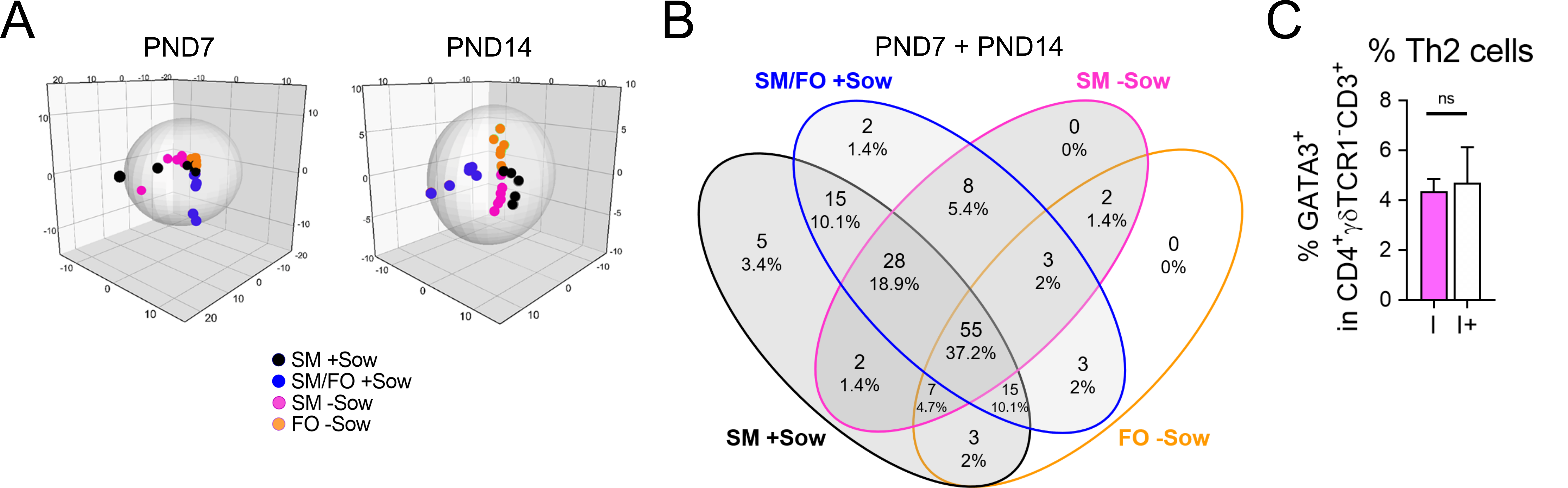

Supplement: Supplementary Figure 6 — Decreased bacterial diversity correlates with a reduction in pulmonary Th1 differentiation, which can be reversed by transfer of material from the maternal environment. (A) Partial Least Squares Discriminant Analysis (PLS-DA) based on the evaluation of the variable importance in projection (VIP) scoring of the 20 most abundant bacterial species in the lungs of 7- and 14-day-old piglets. VIP scoring of bacterial species in the lung is given in Table S1 and S2. (B) Venn diagram showing the distribution of common and unique OTUs in the lungs of 7- and 14-day-old piglets. A total of 132, 129, 105, and 88 different OTUs were identified in SM +Sow, SM/FO +Sow, SM −Sow and FO −Sow groups, respectively. Unique species related to environment, diet, and group are given in Table S3. (C) Bar graph representing the frequencies of Th2 cells in lung. Bars represent the mean value + SD. Statistical analysis was performed by unpaired t test. * P <.05. PND, postnatal day; SM, sow milk; FO, formula; +Sow, sow-reared; −Sow, reared without the sow; I, reared without sow; I+, reared without sow with enrichment. [file Image_6.tif]

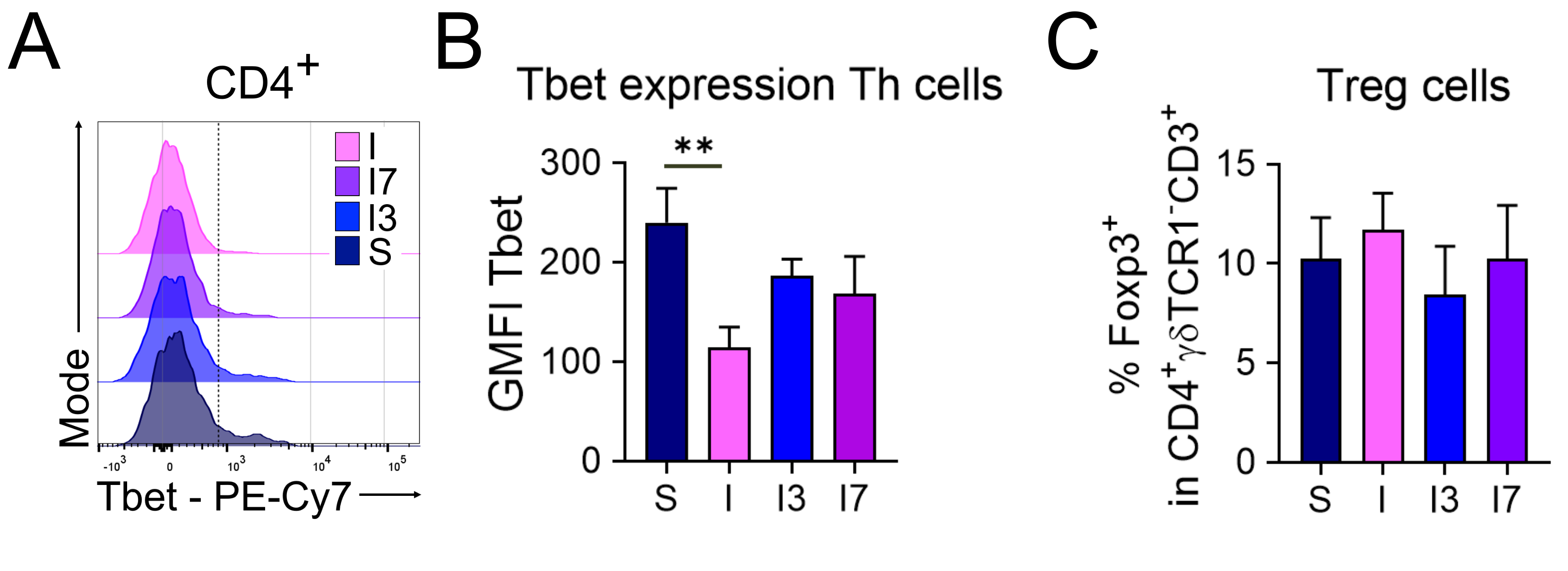

Supplement: Supplementary Figure 7 — Effects of isolation and formula feeding on newborn piglets are reversible within a short interval after birth. (A, B) Representative histograms (A) and bar graph (B) representing the expression level of Tbet in pulmonary Th cells. Bars represent the mean value + SD. Statistical analysis was performed by one-way ANOVA followed by Tukey’s multiple comparisons test. **P <.01. (C) Bar graph representing the frequency of Treg cells in lung. Bars represent the mean value + SD. PND, postnatal day; S, sow-reared; I, reared without sow; I7, reared until PND7 without sow; I3, reared until PND3 without sow; GMFI, geometric mean fluorescence intensity. [file Image_7.tif]

A

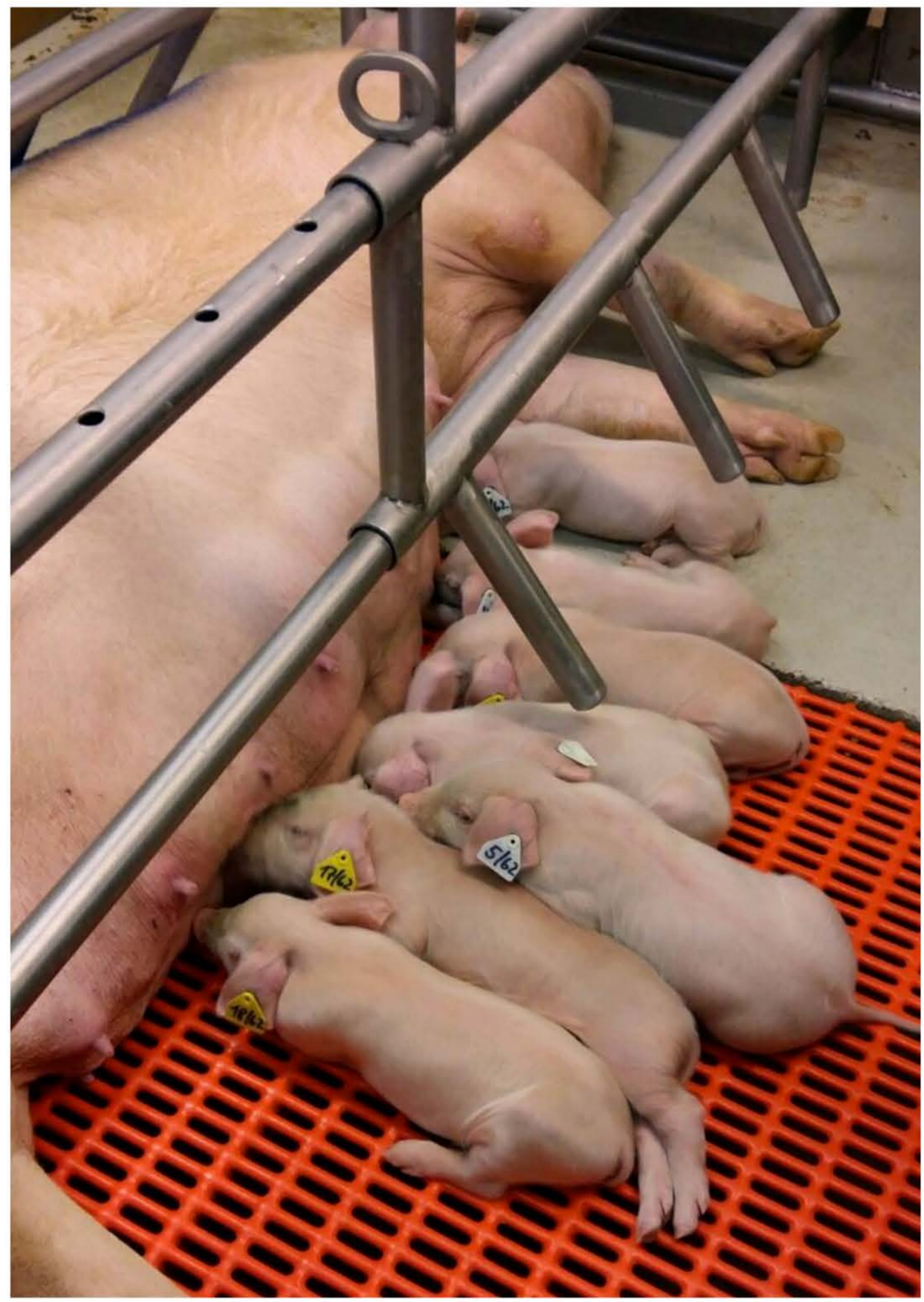

B

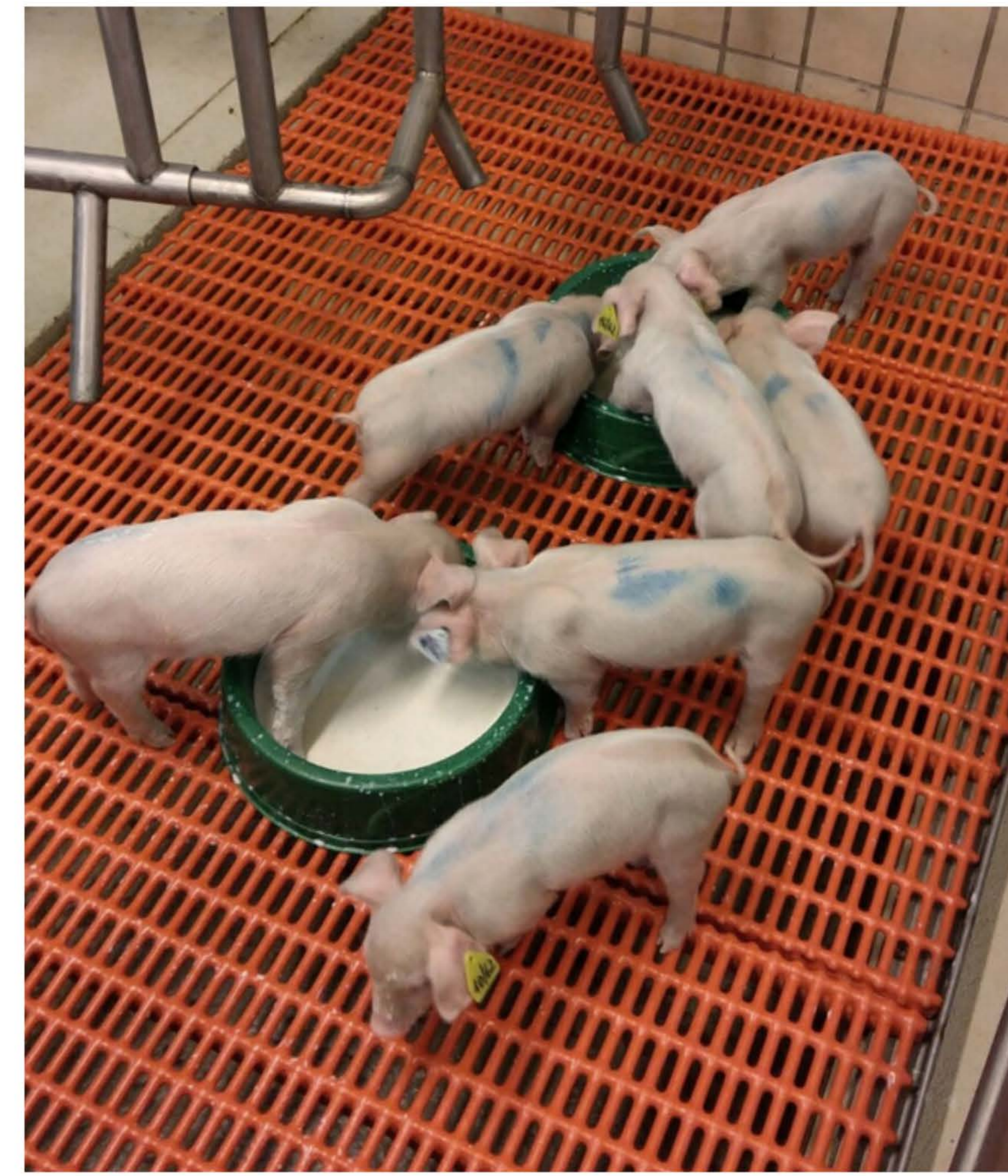

C

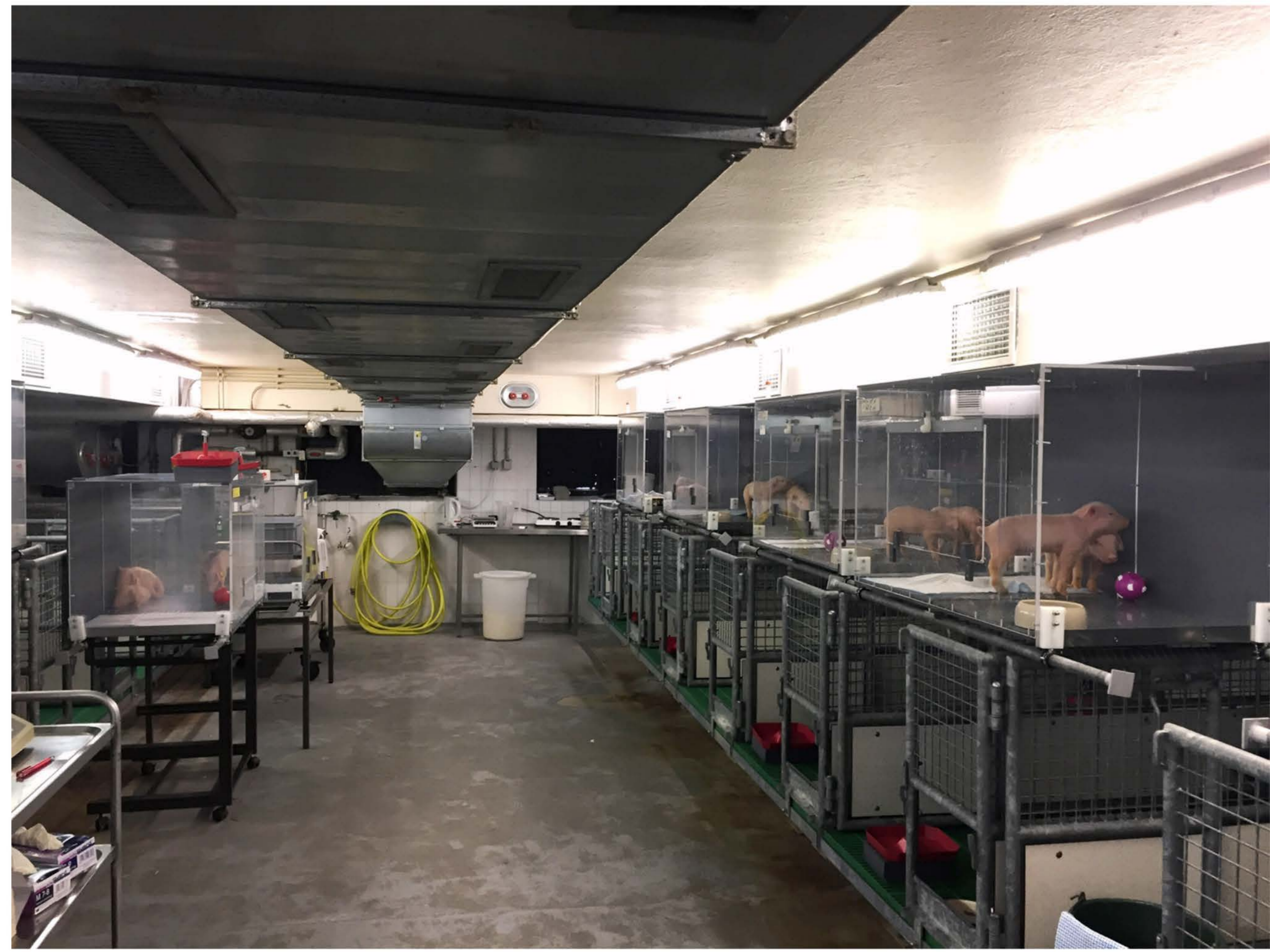

D

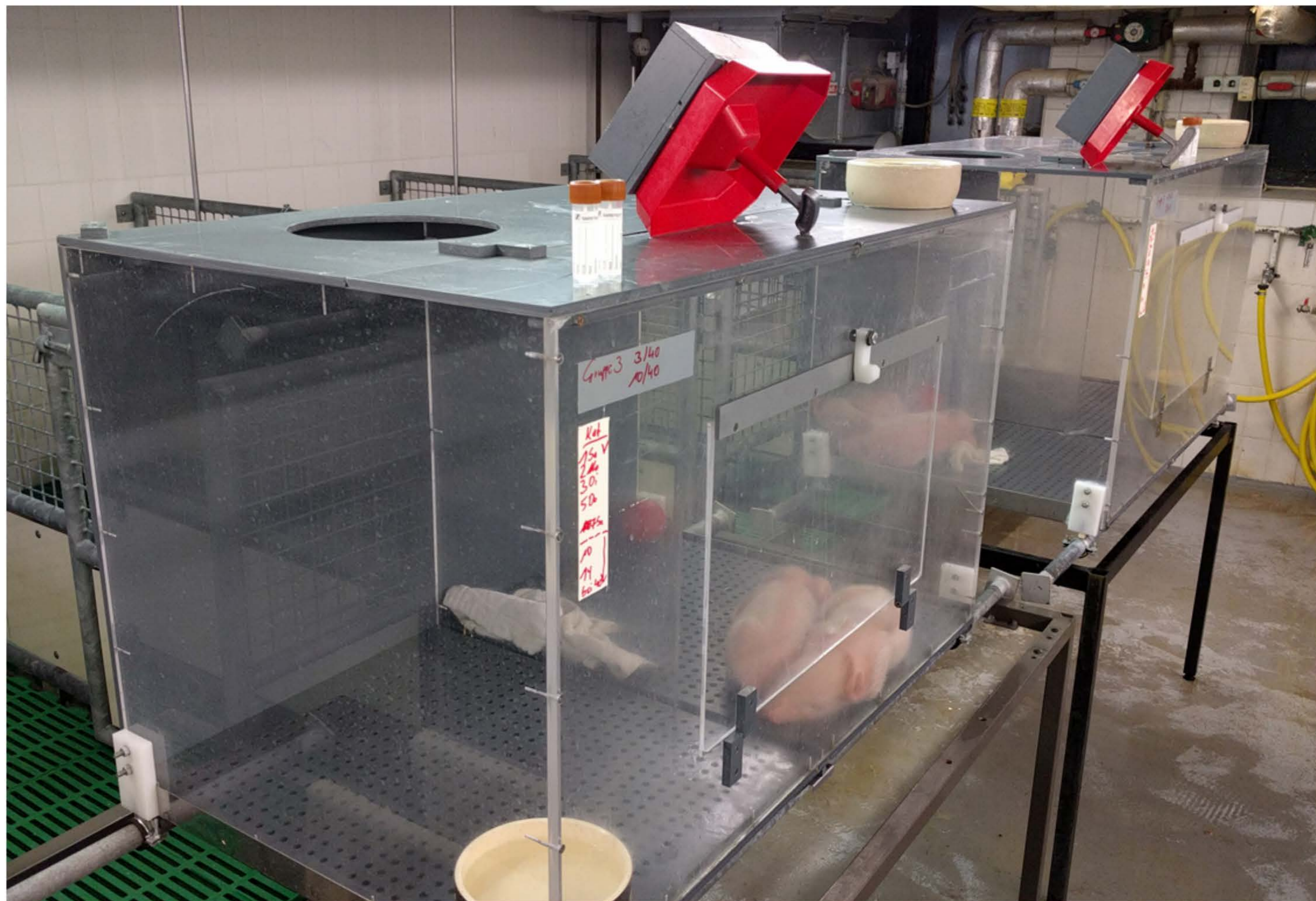

E

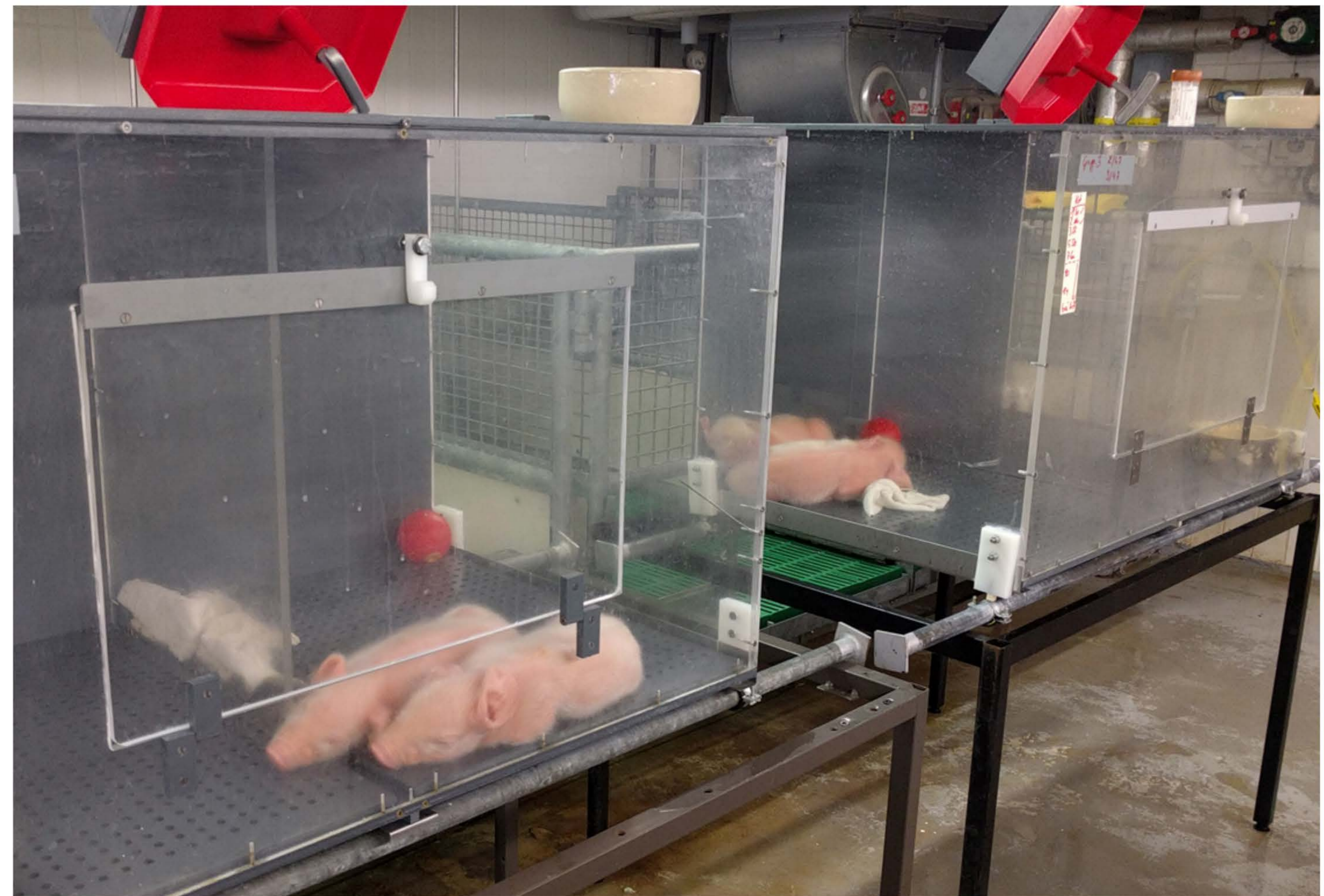

F

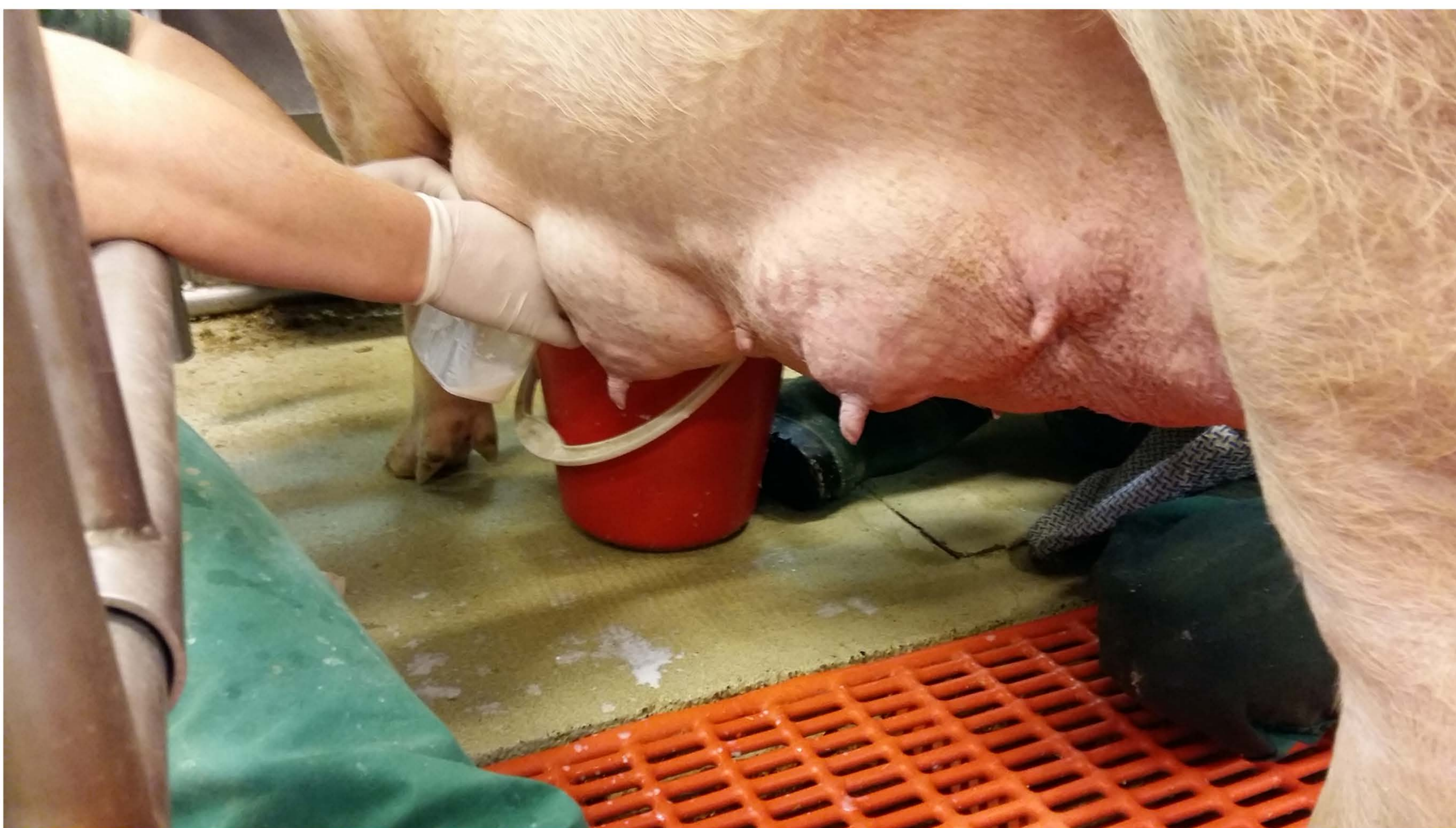

G

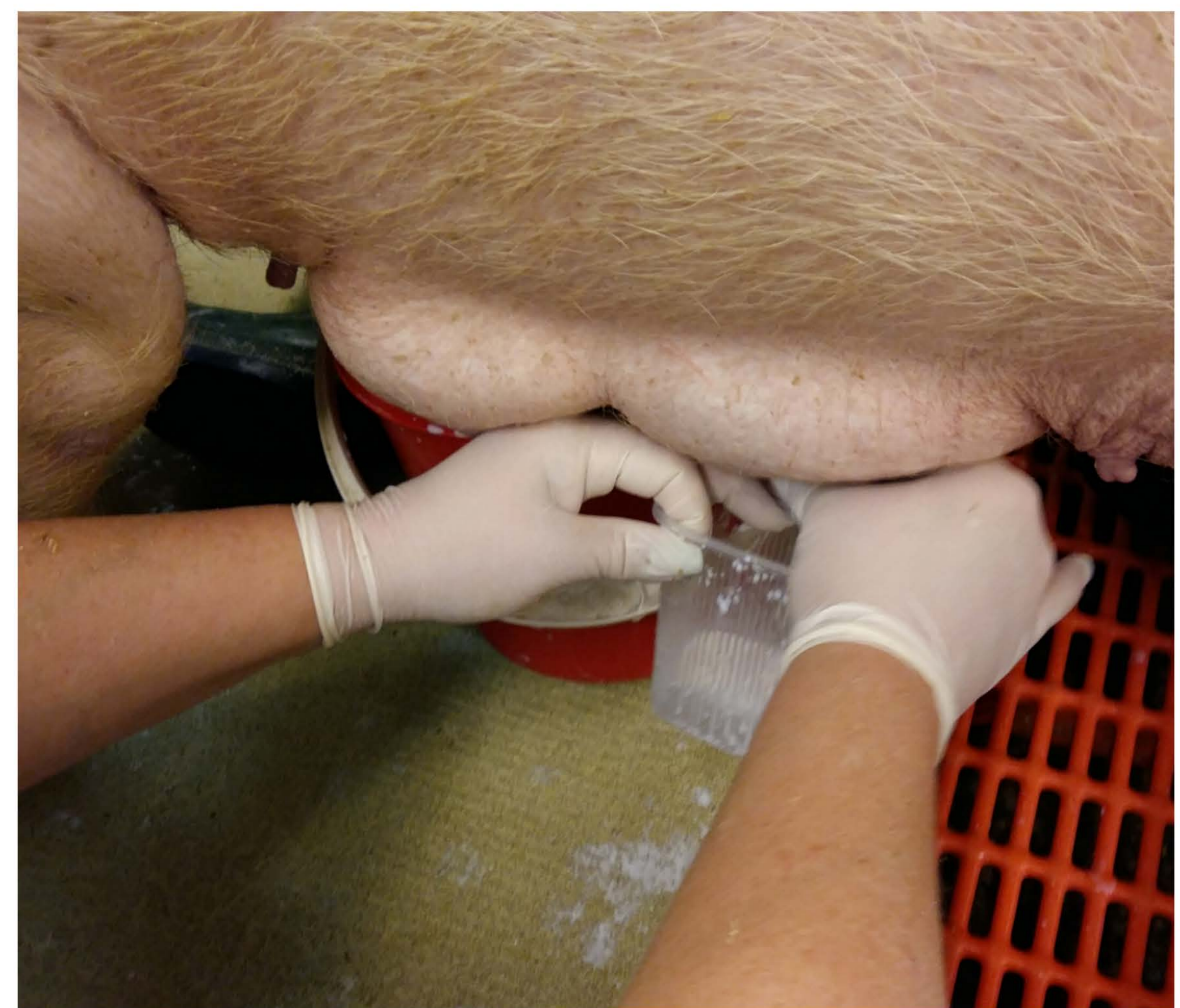

H

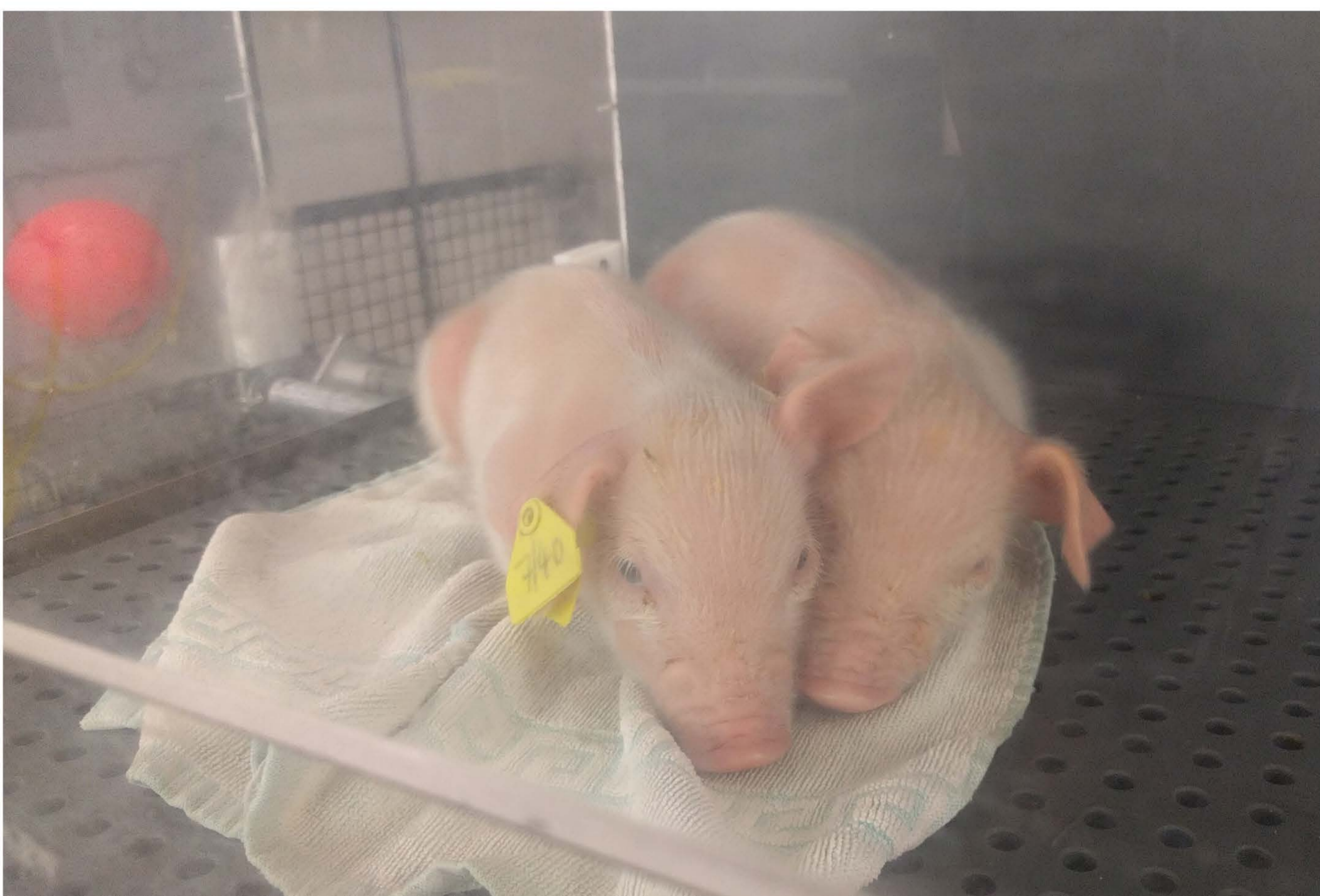

Supplement: Supplementary Figure 8 — Photographs of the experimental setup. (A) Sow-reared piglets. (B) Formula-feeding of sow-reared piglets. (C–E) Rearing units used for sow-deprived piglets. (F, G) Milking of sows by hand. (H) Sow-deprived piglets on a cotton cloth with which the sow and her surroundings were previously wiped. [file Image_8.pdf]
