# Supplementary material for: Influence of Nutrition and Maternal Bonding on Postnatal Lung Development in the Newborn Pig
Source: Front Immunol. 2021 Aug 16;12:734153. doi: 10.3389/fimmu.2021.734153 (PMC8415798; doi:10.3389/fimmu.2021.734153)
Supplement: Supplementary file 9 [file DataSheet_1.docx]

**Supplementary tables**

**Table S1.** Variable Importance in Projection (VIP) scoring of bacterial species in lung swabs of 7-day-old piglets. The table refers to the PLS analysis shown in Figure S6A.

| **Bacterial species** | **VIP score** |
| --- | --- |
| *Prevotella stercorea* | 1.88 |
| *Lactobacillus panis* | 1.84 |
| *Lactobacillus murinus* | 1.84 |
| *Chryseobacterium gleum* | 1.71 |
| *Neisseria canis* | 1.68 |
| *Lactobacillus johnsonii* | 1.59 |
| *Propionibacterium acnes* | 1.59 |
| *Actinomyces naeslundii* | 1.53 |
| *Fusobacterium nucleatum* | 1.49 |
| *Porphyromonas catoniae* | 1.41 |
| *Clostridium botulinum* | 1.37 |
| *Aerococcus viridans* | 1.37 |
| *Lactobacillus delbrueckii* | 1.35 |
| *uncultured proteobacterium* | 1.35 |
| *Capnocytophaga cynodegmi* | 1.35 |
| *Enterococcus faecium* | 1.35 |
| *Propionibacterium freudenreichii* | 1.35 |
| *Lactobacillus ruminis* | 1.34 |
| *Lactobacillus crispatus* | 1.32 |
| *Proteus mirabilis* | 1.31 |
| *Clostridium ramosum* | 1.30 |
| *Actinobacillus porcinus* | 1.29 |
| *Nicoletella semolina* | 1.28 |
| *Staphylococcus delphini* | 1.28 |

**Table S2.** Variable Importance in Projection (VIP) scoring of bacterial species in lung swabs of 14-day-old piglets. The table refers to the PLS analysis shown in Figure S6A.

| **Bacterial species** | **VIP score** |
| --- | --- |
| *Lactobacillus salivarius* | 1.94 |
| *Lactobacillus mucosae* | 1.82 |
| *Streptococcus suis* | 1.72 |
| *Lactobacillus panis* | 1.70 |
| *Fusobacterium periodonticum* | 1.65 |
| *Veillonella dispar* | 1.56 |
| *Prevotella ruminicola* | 1.53 |
| *Prevotella oulorum* | 1.49 |
| *Geitlerinema sp. PCC 7105* | 1.47 |
| *Clostridium perfringens* | 1.46 |
| *Campylobacter jejuni* | 1.46 |
| *Ruminococcus gnavus* | 1.42 |
| *Lactobacillus ultunensis* | 1.41 |
| *Weissella cibaria* | 1.35 |
| *Lactobacillus amylotrophicus* | 1.33 |
| *Veillonella caviae* | 1.31 |
| *uncultured beta proteobacterium* | 1.28 |
| *Bacteroides plebeius* | 1.27 |
| *Rothia mucilaginosa* | 1.27 |
| *Porphyromonas gingivalis* | 1.26 |
| *Lactobacillus johnsonii* | 1.26 |
| *Proteus mirabilis* | 1.25 |
| *Bacteroides vulgatus* | 1.24 |
| *Pseudomonas azotifigens* | 1.20 |

**Table S3.** Unique bacterial species detected in the lung in relation to the environment (sow versus isolated), the diet (sow milk vs. formula) and the four individual experimental groups over the entire experiment. The table refers to the Venn diagram shown in Figure S6B.

| **Group** | **Bacterial species** |
| --- | --- |
| FO -Sow  SM -Sow  SM/FO +Sow  SM +Sow | *Propionibacterium acnes Lactobacillus casei Ruminococcus bromii Ruminococcus gnavus Bacteroides ovatus Bacteroides acidifaciens Bifidobacterium thermacidophilum Fusobacterium periodonticum Butyricimonas virosa Actinobacillus indolicus butyrate-producing bacterium SL7/1 Clostridium scindens Lactobacillus acidophilus Clostridium sphenoides Clostridium disporicum Veillonella caviae Clostridium sordellii Acidaminococcus fermentans Paralactobacillus selangorensis Lactobacillus mucosae Fusobacterium equinum Sarcina ventriculi Clostridium glycolicum Lactobacillus coleohominis Lactobacillus delbrueckii Bacteroides plebeius Lactobacillus helveticus Clostridium bifermentans Staphylococcus hominis Anaerostipes caccae Globicatella sulfidifaciens Lactobacillus gasseri Pseudomonas azotifigens Gemella sanguinis Rothia nasimurium Veillonella dispar Lactobacillus johnsonii Lactobacillus ultunensis Collinsella aerofaciens Blautia sp. Ser8 Butyrivibrio fibrisolvens Lactobacillus salivarius Acetivibrio cellulolyticus uncultured beta proteobacterium Bacteroides fragilis Lactobacillus murinus Lactobacillus reuteri Porphyromonas catoniae Barnesiella intestinihominis Clostridium botulinum Pseudomonas pseudoalcaligenes Prevotella stercorea Lactobacillus vaginalis Haemophilus parasuis Streptococcus suis* |
| SM -Sow  SM/FO +Sow  SM +Sow | *Alistipes finegoldii Actinomyces naeslundii Prevotella albensis Megasphaera elsdenii Parabacteroides distasonis Staphylococcus delphini Aerococcus viridans Clostridium bolteae Fusobacterium nucleatum Macrococcus caseolyticus Actinomyces odontolyticus Streptococcus sp. Enterococcus casseliflavus Acinetobacter lwoffii Veillonella ratti Fusobacterium necrophorum Actinobacillus porcitonsillarum Clostridium spiroforme Lactobacillus crispatus Weissella cibaria uncultured Kingella sp. Ethanoligenens harbinense Neisseria canis Lactobacillus panis Mannheimia varigena Clostridium perfringens Veillonella atypica Micrococcus sp. SMCC ZAT351* |
| FO -Sow  SM/FO +Sow  SM +Sow | *Bacteroides vulgatus Corynebacterium sp. NML96-0244 Selenomonas ruminantium Faecalibacterium prausnitzii Anaerobiospirillum succiniciproducens Clostridium aminophilum Campylobacter mucosalis Actinobacillus porcinus Prevotella copri Lactobacillus amylotrophicus Roseburia intestinalis Pasteurella aerogenes Prevotella baroniae Parabacteroides merdae Bacteroides thetaiotaomicron* |
| FO -Sow  SM -Sow  SM +Sow | *Eubacterium rectale Prevotella veroralis Wautersiella falsenii Clostridium celatum Geitlerinema sp. PCC 7105 Anaeroplasma abactoclasticum Actinobacillus minor* |
| FO -Sow  SM -Sow  SM/FO +Sow | *Nicoletella semolina Ochrobactrum sp. mp-3 Proteus mirabilis* |
| SM/FO +Sow  SM +Sow | *Bacteroides uniformis Clostridium ultunense Prevotella oulorum Streptococcus dysgalactiae Clostridium hylemonae Clostridium cellobioparum Erysipelothrix tonsillarum Dialister pneumosintes Prevotella buccae Staphylococcus haemolyticus Rothia mucilaginosa Prevotella oris Prevotella ruminicola Prevotella bergensis Riemerella anatipestifer* |
| SM -Sow  SM +Sow | *Clostridium thermocellum Chryseobacterium gleum* |
| FO -Sow  SM +Sow | *Corynebacterium freneyi Bacteroides fluxus Staphylococcus saprophyticus* |
| SM -Sow  SM/FO +Sow | *Clostridium ramosum Lactobacillus ruminis Capnocytophaga cynodegmi Lactobacillus curvatus Pediococcus ethanolidurans Propionibacterium acidifaciens uncultured proteobacterium Propionibacterium freudenreichii* |
| FO -Sow  SM/FO +Sow | *Proteus vulgaris Bacteroides massiliensis Bacteroides stercoris* |
| FO -Sow  SM -Sow | *Enterococcus faecium Comamonas aquatica* |
| SM +Sow | *uncultured Klebsiella sp. Actinobacillus rossii Clostridium tertium Bacteroides cellulosolvens Porphyromonas gingivalis* |
| SM/FO +Sow | *Prevotella sp. oral taxon 472 Campylobacter jejuni* |
| SM, sow milk; FO, formula; +Sow, sow-reared and suckling; -Sow, reared isolated from the sow. | |

**Table S4.** Unique bacterial species detected over the entire experiment in the environment (air and room floor), in feces (all piglets) and in the lungs. The table refers to the Venn diagram shown in Figure 6F.

| **Groups** | **Bacterial species** |
| --- | --- |
| Environment  Feces  Lung | *Actinobacillus indolicus Clostridium scindens Actinomyces odontolyticus Fusobacterium necrophorum Lactobacillus delbrueckii Blautia sp. Ser8 Bacteroides fragilis Bacteroides ovatus Fusobacterium nucleatum Bacteroides plebeius Lactobacillus helveticus Lactobacillus murinus Clostridium perfringens Prevotella oulorum Bifidobacterium thermacidophilum Corynebacterium sp. NML96-0244 Clostridium hylemonae Faecalibacterium prausnitzii Clostridium bolteae Clostridium glycolicum Collinsella aerofaciens Lactobacillus salivarius Lactobacillus reuteri Streptococcus suis Paralactobacillus selangorensis Lactobacillus mucosae Ethanoligenens harbinense Roseburia intestinalis Parabacteroides merdae* |
| Feces  Lung | *Bacteroides uniformis Lactobacillus casei Clostridium thermocellum Ruminococcus bromii Prevotella sp. oral taxon 472 Enterococcus faecium Staphylococcus delphini Selenomonas ruminantium Clostridium cellobioparum Veillonella caviae Clostridium sordellii Clostridium aminophilum Bacteroides massiliensis Clostridium spiroforme Bacteroides cellulosolvens Rothia nasimurium Lactobacillus ultunensis Prevotella baroniae Mannheimia varigena Anaeroplasma abactoclasticum Propionibacterium acidifaciens Actinobacillus minor Veillonella atypica Bacteroides thetaiotaomicron Haemophilus parasuis Prevotella veroralis Bacteroides fluxus Ruminococcus gnavus Megasphaera elsdenii Streptococcus dysgalactiae Parabacteroides distasonis butyrate-producing bacterium SL7/1 Lactobacillus acidophilus Clostridium disporicum Macrococcus caseolyticus Actinobacillus rossii Actinobacillus porcinus Actinobacillus porcitonsillarum Clostridium tertium Staphylococcus hominis Bacteroides stercoris Globicatella sulfidifaciens Weissella cibaria Lactobacillus gasseri Butyrivibrio fibrisolvens Prevotella ruminicola Clostridium botulinum Prevotella stercorea Riemerella anatipestifer Corynebacterium freneyi Eubacterium rectale Alistipes finegoldii Clostridium ramosum Fusobacterium periodonticum Aerococcus viridans Erysipelothrix tonsillarum Acidaminococcus fermentans Lactobacillus ruminis Streptococcus sp. Prevotella copri Clostridium bifermentans Lactobacillus curvatus Anaerostipes caccae Lactobacillus crispatus Porphyromonas gingivalis Prevotella oris Proteus mirabilis Pasteurella aerogenes Acetivibrio cellulolyticus Lactobacillus panis Anabaena sp. BIR361 Lactobacillus vaginalis Clostridium ultunense Actinomyces naeslundii Prevotella albensis Bacteroides vulgatus Bacteroides acidifaciens Staphylococcus saprophyticus Butyricimonas virosa Clostridium sphenoides Clostridium celatum Campylobacter jejuni Dialister pneumosintes Anaerobiospirillum succiniciproducens Enterococcus casseliflavus Prevotella buccae Veillonella ratti Fusobacterium equinum Sarcina ventriculi Lactobacillus coleohominis Staphylococcus haemolyticus Lactobacillus amylotrophicus Gemella sanguinis Veillonella dispar Lactobacillus johnsonii Pediococcus ethanolidurans Porphyromonas catoniae Barnesiella intestinihominis Prevotella bergensis Micrococcus sp. SMCC ZAT351* |
| Environment  Lung | *Propionibacterium acnes Acinetobacter lwoffii Comamonas aquatica Pseudomonas azotifigens* |
| Lung | *Nicoletella semolina uncultured Kingella sp. Pseudomonas pseudoalcaligenes uncultured proteobacterium Propionibacterium freudenreichii uncultured beta proteobacterium Wautersiella falsenii Proteus vulgaris Campylobacter mucosalis Ochrobactrum sp. mp-3 Rothia mucilaginosa Neisseria canis Geitlerinema sp. PCC 7105 Chryseobacterium gleum Capnocytophaga cynodegmi* |
| SM, sow milk; FO, formula; +Sow, sow-reared and suckling; -Sow, reared isolated from the sow. | |
